# Characterization of fully-evaporated perovskite solar cells and photodetectors under high-intensity pulsed proton irradiation

**DOI:** 10.1038/s41598-024-67541-5

**Published:** 2024-07-20

**Authors:** Hryhorii P. Parkhomenko, Andriy I. Mostovyi, Marat Kaikanov, Jessica Strey, Mircea C. Turcu, Marvin Diederich, Sascha J. Wolter, Verena Steckenreiter, Joachim Vollbrecht, Viktor V. Brus

**Affiliations:** 1 Department of Physics, School of Sciences and Humanities, Nazarbayev University, Astana, 010000 Republic of Kazakhstan; 2 Department of Photovoltaics, Institute for Solar Energy Research Hamelin, 31860 Emmerthal, Germany

**Keywords:** Perovskite solar cells, Perovskite photodetector, Radiation hardness, Proton irradiation, Recombination dynamics, Optical sensors, Optoelectronic devices and components, Solar energy and photovoltaic technology

## Abstract

This study investigates the impact of proton irradiation on perovskite devices fabricated fully through vacuum deposition. Exposure to irradiation induces changes in both electrical and optical properties. The analysis reveals that the main factors influencing the observed performance changes in solar cells are a significant reduction in shunt resistance and a minor increase in series resistance, with minimal alterations in recombination dynamics. Remarkably, the devices maintain promising photodetector characteristics both before and after proton irradiation, particularly in a self-powered mode without a reverse bias. These findings provide valuable insights into the resilience of vacuum-deposited perovskite devices against ionizing radiation, highlighting their potential for applications in radiation-prone environments, such as the nuclear industry or space exploration.

## Introduction

In recent years, the field of hybrid inorganic–organic photovoltaic devices has witnessed remarkable advancements, particularly in the realm of active layers based on perovskites^1–4^. These developments have led to notable achievements, with independently reported power conversion efficiencies surpassing *η* = 26.1% in single-junction perovskite solar cells (PSCs) and the efforts to combine the advantages of PSCs with those exhibited by silicon solar cells resulted in perovskite/Si-tandem devices with record-breaking efficiencies (*η* = 33.9%)^5^. However, the quest for high efficiencies is not the sole focus of current PSC research; it has also extended to encompass other critical areas: reproducibility, long-term stability, and the facilitation of large-area deposition techniques^6–12^.

In parallel with these endeavors, the utilization of perovskites as active layers in various optoelectronic devices has garnered significant attention in the recent years. Applications such as light-emitting diodes (LEDs) and photodetectors (PDs) have emerged as notable use-cases^13–15^. In photodetectors, specific device attributes have assumed paramount importance, including the capability to detect photons across a defined wavelength range over several orders of magnitude of illumination intensities and the operational speeds of the devices^16,17^. Additionally, the ability to withstand environmental stressors has become a crucial consideration for PD applications. Particularly noteworthy is the suitability of perovskite materials for scenarios necessitating enhanced radiation hardness, such as in the nuclear industry or space exploration^18–21^.

The latter example has recently been in the spotlight, since deposition of perovskite materials, along with other essential layers such as transport layers and metal electrodes, can be effectively achieved through evaporation techniques^22,23^. This method not only offers precise control over layer deposition but also presents a compelling advantage for in-situ device fabrication in space applications. The vacuum conditions required for evaporation, though costly on Earth, naturally align with the vacuum conditions prevalent in the space environment, making this fabrication approach a preferred choice for advancing PSC and PD technology in space^24–27^.

In particular, the atmospheric pressure at the Kármán line (100 km altitude) drops to *p* ≈ 10^−4^ mbar and at low Earth orbit (500 km altitude) to *p* ≈ 10^−10^–10^−8^ mbar; as a point of reference, the highest pressure still considered to be ultra-high vacuum is *p* = 10^−9^ mbar, which is orders of magnitude below the processing pressures for most evaporation techniques^28–30^.

**Figure 1:**
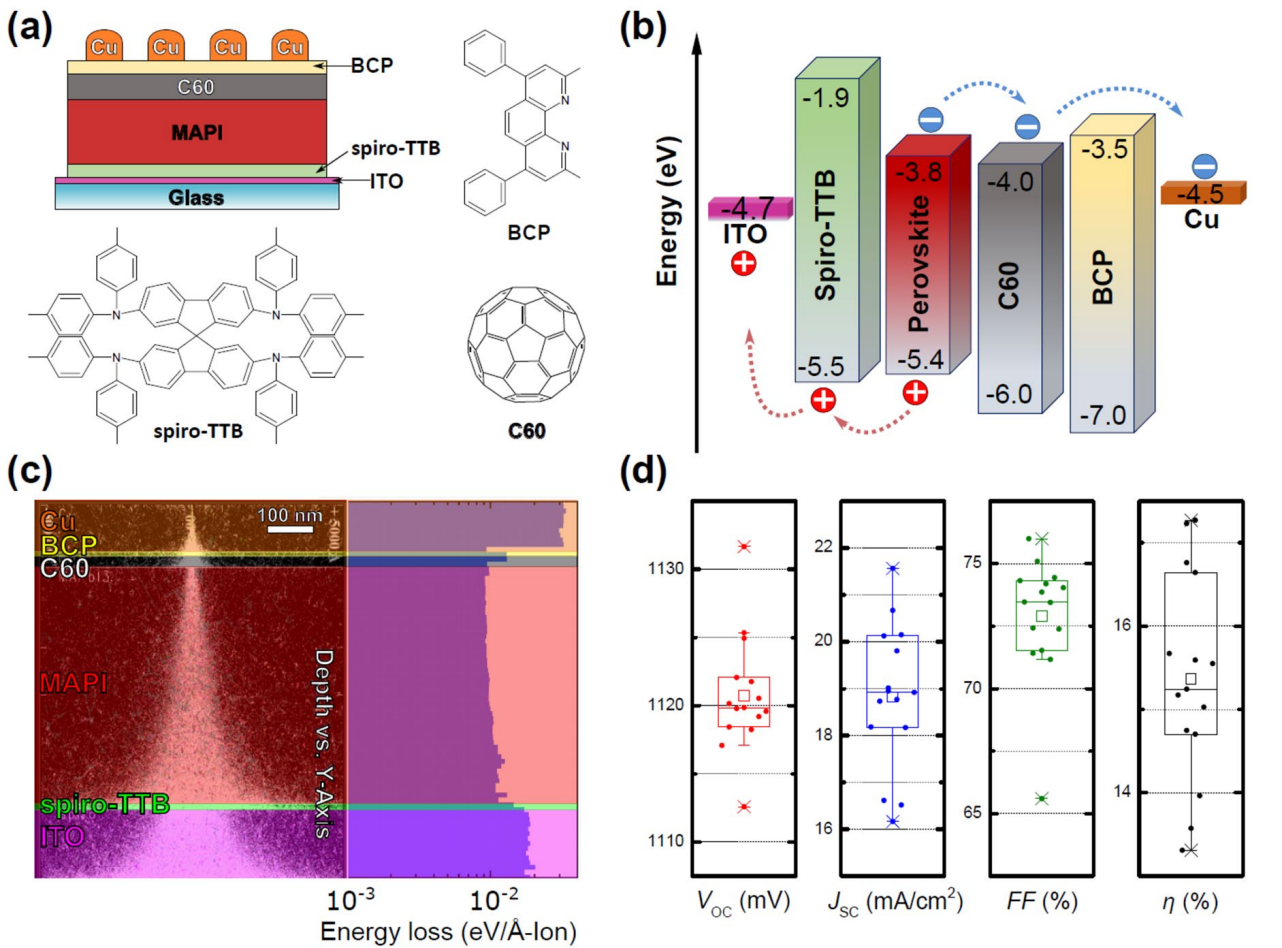
(**a**) Schematic depiction, chemical structures, (**b**) energy levels, and (**c**) SRIM simulations of the studied MAPI devices. (**d**) Initial open-circuit voltage (*V*_OC_), short-circuit current density (*J*_SC_), fill factor (*FF*), and power conversion efficiency (*η*) of the tested solar cells (*n* = 15).

In this study, we employed a complete vacuum deposition process to fabricate methylammonium lead triiodide (CH_3_NH_3_PbI_3_; MAPI) based perovskite devices featuring the layer sequence glass/ITO/spiro-TTB/CH_3_NH_3_PbI_3_/C60/BCP/Cu (cf. Fig. 1, ESI). As the initial operational characteristics, values up to an open-circuit voltage *V*_OC_ = 1131.66 mV, short-circuit current density *J*_SC_ = 21.56 mA cm^−2^, fill factor *FF* = 75.99%, and power conversion efficiency *η* = 17.27% were achieved; further details are described in Table 1 and Fig. S1. Following this, the devices underwent irradiation with protons via three short pulses (150 ns) of 2 × 10^12^ p cm^−2^, at an energy of 140 keV, imparting a total fluence of 6 × 10^12^ p cm^−2^. As a point of reference, a fluence of 10^13^ p cm^−2^ is usually accumulated over a duration of roughly 30 years at low Earth orbit outside the Van-Allen belts^31^. Subsequent to proton irradiation, comprehensive assessments of the devices as both solar cells and photodetectors were conducted, facilitating a thorough comparison of their performance before and after exposure to ionizing radiation (cf. ESI).

**Table 1:** Photovoltaic parameters obtained from the *J*–*V-*characteristics of the tested devices.

Sample	*V*_OC_ [mV]	*J*_SC_ [mA cm^−2^]	*FF* [%]	*η* [%]
Initially*	1120.75 ± 4.29 (1131.66)	18.82 ± 1.55 (21.56)	72.89 ± 2.45 (75.99)	15.36 ± 1.55 (17.27)
Before irradiation	1078.11	17.17	72.38	13.43
After irradiation	1055.77	12.00	56.36	7.14

*Average values of 15 tested devices and their standard deviation, maximum values listed in brackets (cf. Fig. S1).

## Results and discussion

The damage profile and penetration depth of protons in the device were determined using stopping and range ion modeling (SRIM) utilizing experimentally measured thicknesses and densities of functional layers taken from the literature^32,33^ and SRIM libraries. Protons with an energy of 140 keV penetrate all functional layers of the device with decent energy losses for recoils (cf. Fig. 1c), indicating optimal conditions for testing radiation resistance^34,35^. Damage to the functional layers (ETL, HTL, metal, and transparent electrode) will primarily impact the series resistance of the devices, as the resistance of the layers may increase during the irradiation process^19^. The alterations induced by proton irradiation in the perovskite active layer are crucial since the generation-recombination processes occurring in the active layer play a pivotal role in the operation of optoelectronic devices^36–38^.

Our study entails a thorough examination of device performance before and after exposure to irradiation. Two key parameters are the cornerstone of the subsequent analyses: the light intensity-dependent voltage–current–density (*J*–*V*) characteristics and the external quantum efficiency (*EQE*). These metrics are crucial for assessing the functionality of the devices, particularly in PSC and PD applications.

The photovoltaic parameters derived from the *J*–*V*-characteristics measured initially after fabrication, directly before irradiation, and directly after irradiation are listed in Table 1. It should be noted that the behavior of the *J–V* curves of perovskite optoelectronic devices is dependent on the architecture of the device, pre-bias conditions, and pre-conditioning cycles^39,40^. In the present case, the *J*–*V*-characteristics were measured without the use of pre-bias and pre-conditioning cycles. The reduction between the initial testing of the newly fabricated devices and before irradiation can be linked to performance losses resulting from transporting the devices across continents.

A relatively small reduction in *V*_OC_ as well as more significant reductions in *J*_SC_ and *FF* can be observed after the tested devices have been subjected to the irradiation treatment. Unsurprisingly, the latter two parameters are the main reason for the reduction of the power conversion efficiency from *η* = 13.43% to *η* = 7.14%.

**Figure 2:**
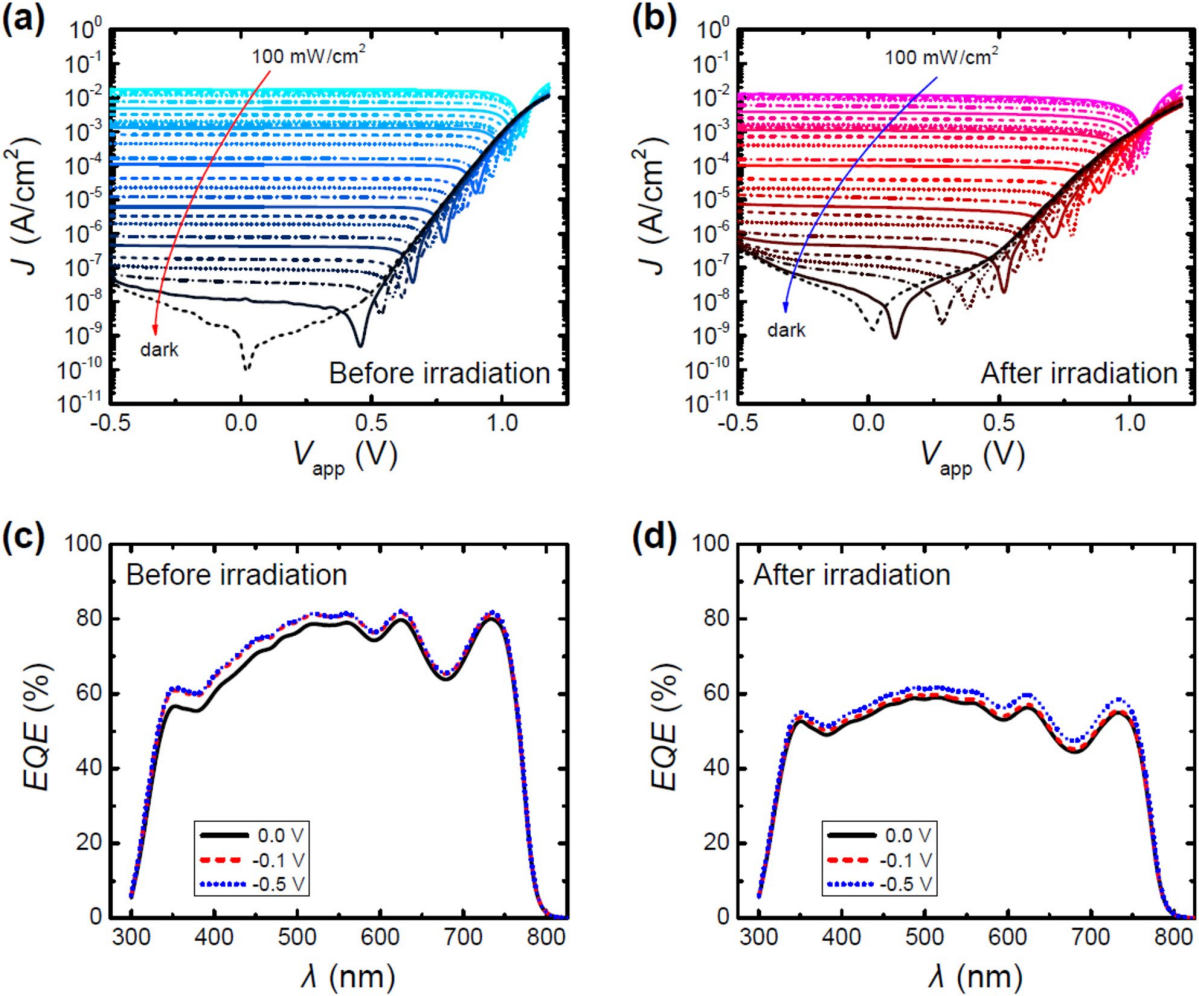
(**a**,**b**) Light intensity dependent *J*–*V*-characteristics of the tested devices before and after irradiation. (**c**,**d**) External quantum efficiency at different applied voltages of the tested devices before and after irradiation.

The *J*–*V*-characteristics were measured across a broad range of light intensities, providing foundational data for in-depth device evaluation and subsequent intricate analyses of device behavior (cf. Fig. 2). Even when subjected to low illumination intensities, we observed that the changes in current density at reverse and low forward biases remained relatively small, suggesting that the devices exhibited stability under these conditions. However, a notably different behavior in the current–density profile became apparent after irradiation. Specifically, we observed a more pronounced bias-dependent increase in the absolute value of current density at low illumination intensities.

The external quantum efficiency (*EQE*) measurements of the devices were essential in understanding their response to irradiation, particularly in the context of their spectral sensitivity (cf. Fig. 2). Prior to irradiation, the *EQE* measurements showed increased values in the wavelength region of *λ* = 350–600 nm when a reverse bias was applied. After irradiation, the *EQE* landscape exhibited significant changes. Notably, there was a reduction in *EQE*, primarily in long wavelength ranges (*λ* = 500–750 nm). Intriguingly, the bias-driven increase in *EQE* observed before irradiation in the *λ* = 350–600 nm range was less pronounced post-irradiation, with the remaining bias-driven enhancement being now primarily centered in the *λ* = 500–750 nm range.

The observed changes in the *J*–*V*-characteristics and *EQE* provide valuable insights into the impact of irradiation on the devices’ performance. The increase in current density after irradiation suggests alterations in the electrical properties of the device, which may be attributed to irradiation-induced defects or changes in carrier mobility. The reduction in *EQE* at longer wavelengths after irradiation could indicate a shift in the absorption properties of the devices, possibly due to structural modifications caused by irradiation. The change in *EQE* enhancement from the *λ* = 350–600 nm range to the *λ* = 500–750 nm range suggests a spectral reconfiguration of the sensitivity of the devices.

### Solar cells

**Figure 3:**
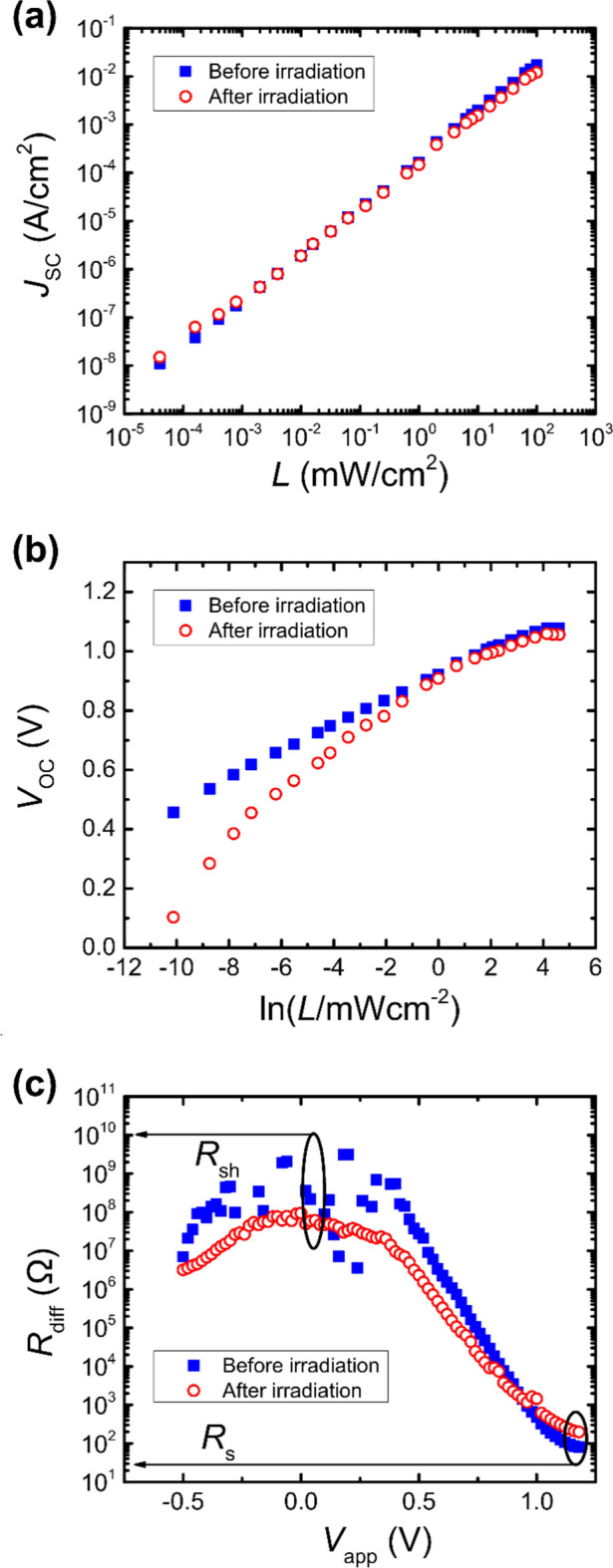
(**a**) Light intensity-dependent short-circuit current density and (**b**) open-circuit voltage of the tested devices before and after irradiation. (**c**) Differential resistance of the tested devices before and after irradiation.

A more advanced analysis of the tested devices as PSCs is based on the qualitative and quantitative analysis of the recombination dynamics before and after irradiation. The qualitative analysis is spear headed by the characterization of the light intensity dependent *J*_SC_ and *V*_OC_ behavior (cf. Fig. 3)^41–43^. The *J*_SC_ of the tested devices exhibits a linear relationship over a wide light intensity range in a double-logarithmic plot:

1 $$ J_{SC} = L^{\alpha } \Leftrightarrow log\;J_{SC} = \alpha \;log\;L. $$

The resulting proportionality factor of *α* = 0.9834 and *α* = 0.9395 for the devices before and after irradiation, respectively, are indicative of an increased band-to-band recombination, once the devices were irradiated.

In contrast, the open-circuit voltages exhibit more drastic and significant changes over the tested light intensity range, including even more pronounced differences of the tested devices before and after irradiation. A semi-logarithmic relationship between the *V*_OC_ and the light intensity is employed to estimate the dominant type of recombination mechanism:

2 $$ V_{OC} \propto S\frac{kT}{q} \cdot \ln L $$

where *k* is the Boltzmann constant, *T* is the absolute temperature, *q* is the elementary charge, and *S* is a unitless factor that indicates the dominant type of recombination mechanism. Dominant band-to-band recombination yields *S* = 1, bulk-trap assisted recombination leads to *S* > 1, whereas surface-trap assisted recombination results in *S* < 1^44–47^. The devices before irradiation tend to yield a linear behavior in the semi-logarithmic plot, whereas the irradiated devices exhibit non-linear properties, specifically at lower light intensities. This latter observation is likely linked with a reduced shunt resistance *R*_sh_ of the irradiated devices, which usually result in non-linear behavior of the *V*_OC_ at low light intensities. This is verified by the determination of the differential resistance (*R*_diff_ = d*I*/d*V*) based on the *I*-*V*-curves in the dark. At high forward bias, the differential resistance can be assumed to be equal to the series resistance *R*_s_, whereas the shunt resistance *R*_sh_ can be estimated from the differential resistance at an applied voltage of *V*_app_ = 0 V^48^. Indeed, a significant reduction of the shunt resistance can be observed, once the tested PSCs were irradiated, namely from *R*_sh_ = 509 MΩ to *R*_sh_ = 94 MΩ. Conversely, an increased series resistance was exhibited by the tested devices, once they were irradiated, namely from *R*_s_ = 79.3 Ω to *R*_s_ = 197.2 Ω, respectively.

Nonetheless, the evaluation of the slope factor *S* in the linear parts of the *V*_OC_–ln*L*-plot reveals that the recombination dynamics is predominantly band-to-band recombination, regardless of whether the PSCs were irradiated or not. However, the possibility exists that the impact of bulk and surface trap-assisted recombination contributions might counterbalance each other, resulting in slopes close to 1*kT*/*q*^49,50^. Therefore, it was necessary to perform more elaborate experiments to elucidate quantitatively the recombination dynamics of these devices. A combination of open-circuit-voltage-decay (OCVD) and impedance spectroscopy (IS) measurements yielded important insights regarding precisely the quantitative recombination dynamics. The transient nature of the OCVD technique facilitated the observation of the voltage changes of the tested device, which is held at open-circuit conditions by employing a high-impedance buffer, after turning off the external illumination source^51^. While OCVD on its own can already provide detailed information regarding the recombination dynamics, its full potential is unlocked, if it is combined with another technique that allows to convert the transient open-circuit voltage *V*_OC_(*t*) into the transient charge carrier density *n*_OC_(*t*)^45,46^. In this study, impedance spectroscopy was employed to determine the charge carrier density under open-circuit conditions and the same illumination conditions that were used for the OCVD measurements. Thus, a direct link between the voltage *V*_OC_ (*t* = 0) and the charge carrier density *n*_OC_ could be established (cf. Figs. S3 and S4)^48,52–54^.

Similar to the qualitative investigation of the recombination dynamics via the light-intensity dependent *V*_OC_, three types—first, second, and third order processes—of possible recombination pathways were taken into consideration during the quantitative analysis that is based on the experimental results obtained from OCVD and IS. It is revealed that all aforementioned types of recombination contribute to the total recombination rate (cf. Fig. S5 and Table 2). Interestingly, the irradiation leads to an increase of the first order recombination coefficient by nearly one order of magnitude, namely from *k*_1_ = 5.69 s^−1^ to *k*_1_ = 49.81 s^−1^. The band-to-band recombination also increases after irradiation, as evidenced by the relevant coefficient changing from *k*_2_ = 1.85 × 10^−14^ cm^3^·s^−1^ to k_2_ = 3.26 × 10^−14^ cm^3^ s^−1^. The contributions from third or pseudo-third order recombination, which in all likelihood are stemming from surface trap-assisted recombination rather than Auger recombination, remain on a similar level before and after irradiation.

**Table 2:** Relevant parameters describing the recombination and loss processes of the tested devices.

Sample	*α*	*S* [*kT*/*q*]	*R*_sh_ [MΩ]	*R*_s_ [Ω]	*k*_1_ [s^−1^]	*k*_2_ [cm^3^ s^−1^]	*k*_3_ [s^−1^]
Before irradiation	0.9834	1.18	509.0	79.3	5.69	1.85 × 10^−14^	1.50 × 10^−11^
After irradiation	0.9395	1.09	94.4	197.2	49.81	3.26 × 10^−14^	1.18 × 10^−11^

Overall, the analysis as PSCs of the tested devices before and after proton irradiation indicates that the main contributing factors for the observed changes in performance are the significant reduction of the shunt resistance *R*_sh_ and to a lesser extend the increased series resistance *R*_s_. While the qualitative and quantitative analysis of the recombination dynamics revealed some changes upon irradiation, these appear to be only of minor consequence. However, it is known that an increase in charge carrier recombination can result in a reduction in shunt resistance and, accordingly, an increase in the leakage current^55–57^. This increase in leakage currents is responsible for the nonlinear nature of the light intensity-dependent open-circuit voltage discussed above^58^. Furthermore, the decrease in the open-circuit voltage after irradiation may also be due to an increase in recombination losses^20,53^. The observed increase in series resistance of the PSCs subsequent to proton irradiation is primarily attributed to damage to the functional layers (ETL/HTL and/or electrodes)^20,59^.

Moreover, the MAPI devices investigated in this study demonstrate pre-irradiation and post-irradiation efficiencies that rank among the highest reported for MAPI devices subjected to analogous irradiation treatments in the existing literature. However, it is noteworthy that perovskite compositions with more intricate structures generally maintain superior efficiencies following proton irradiation of comparable energies and fluences (cf. Table S1).

### Photodetectors

**Figure 4:**
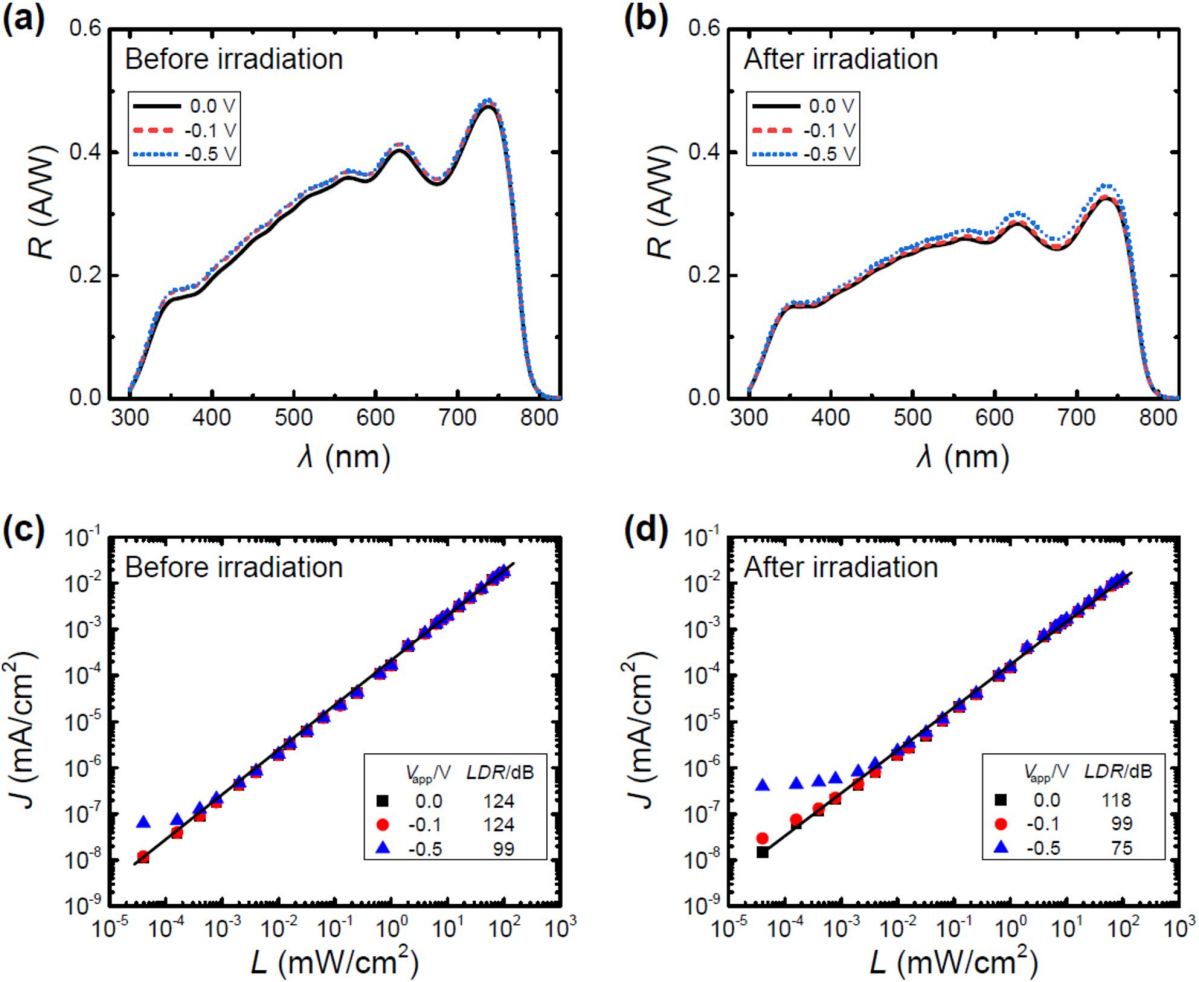
(**a**,**b**) Responsivity at different applied voltages of the tested devices before and after proton irradiation. (**c**,**d**) Determination of the linear dynamic range (*LDR*) at different applied voltages of the tested devices before and after proton irradiation. The solid black line acts as a guide to the eye.

The operation of these perovskite devices as photodetectors requires a thorough examination of two aspects that only in part align with the analyses that are usually employed to study PSCs. The first important property that is commonly tested for devices that are used as PDs is the responsivity *R*. The responsivity of PDs is used as a figure-of-merit that characterizes the ratio of the electrical output in relation to the optical input. In units of ampere produced per watt of incident light, *R* can be calculated as follows^19^:

3 $$ R = \frac{{J_{ph} }}{L} = \frac{EQE}{{100\% }} \cdot \frac{\lambda }{{1240\left( {nm \cdot W \cdot A^{ - 1} } \right)}}, $$

where *J*_ph_ is the photocurrent density, *L* is the incident light intensity, *λ* is the wavelength of the incident light in nanometers, and *EQE* is the external quantum efficiency. In the case of the tested perovskite devices, it can be seen that the responsivity *R* before proton irradiation exhibits its maximum of *R* = 0.48 A/W in the region of *λ* = 750 nm (cf. Fig. 4). Furthermore, only a small increase of the responsivity *R* occurs, if a reverse bias is applied. After proton irradiation, a reduction of the responsivity can be observed, most notably in the region of *λ* = 500–750 nm, which is directly linked to the changes in the *EQE* when comparing the devices before and after proton irradiation (cf. Fig. 2). Consequently, only a maximum of *R* = 0.35 A/W can be achieved. Similarly, the irradiated devices also exhibit a more pronounced reverse bias-driven enhancement of the responsivity *R* in the wavelength range that suffered the most significant responsivity reduction.

The second critical parameter is the linear dynamic range (*LDR*). This metric characterizes the extent over which the response of the tested PDs exhibits a linear relationship with the incident signal. The *LDR*, expressed in decibels (dB), can be formally defined and calculated as follows^20^:

4 $$ LDR = 20\;log\frac{{L_{u} }}{{L_{l} }} = 20\;log\frac{{J_{u} }}{{J_{l} }}, $$

Here, the variables *L*_u_ and *L*_l_ represent the upper and lower irradiance levels, respectively. These values delineate the boundaries beyond which deviations from linearity in the signal-irradiance relationship of the PD become noticeable. Consequently, the corresponding photocurrents are denoted as *J*_u_ and *J*_l_, respectively.

In the case of the tested perovskite PDs, the linear dynamic range reached values of *LDR* = 124 dB for the device before proton irradiation, without reverse bias as well as with an applied bias of *V*_app_ = − 0.1 V. The linear dynamic range decreased to *LDR* = 99 dB, once a stronger reverse bias of *V*_app_ = − 0.5 V was applied. A reduction of the *LDR* was also observed after proton irradiation. This effect was only moderate without any reverse bias (*LDR* = 118 dB), but more pronounced for the cases, where a reverse bias of *V*_app_ = − 0.1 V or *V*_app_ = − 0.5 V was applied (*LDR* = 99 dB and *LDR* = 75 dB, respectively). The deviation from linearity consistently occurs at lower light intensities across all tested conditions, aligning with the previously discussed behavior of *J*–*V*-curves at low light levels. Specifically, this behavior is characterized by an increase in current density attributed to an intensified absolute reverse bias. Hence, this observation is directly linked to the property of the tested devices to act as diodes blocking current flow. Moreover, exposure to proton irradiation led to a further increase in current density within this bias region, which indicates that proton irradiation has a negative impact on the diode characteristics of the tested devices, which agrees with the observed reduction in the shunt resistance *R*_sh_ discussed previously. In contrast, there were no deviations from linearity at light intensities up to and including *L* = 100 mW cm^−2^. Under the aforementioned circumstances, the preferred operational mode of the tested PDs is a self-powered one or in other words without the application of a reverse bias.

**Figure 5:**
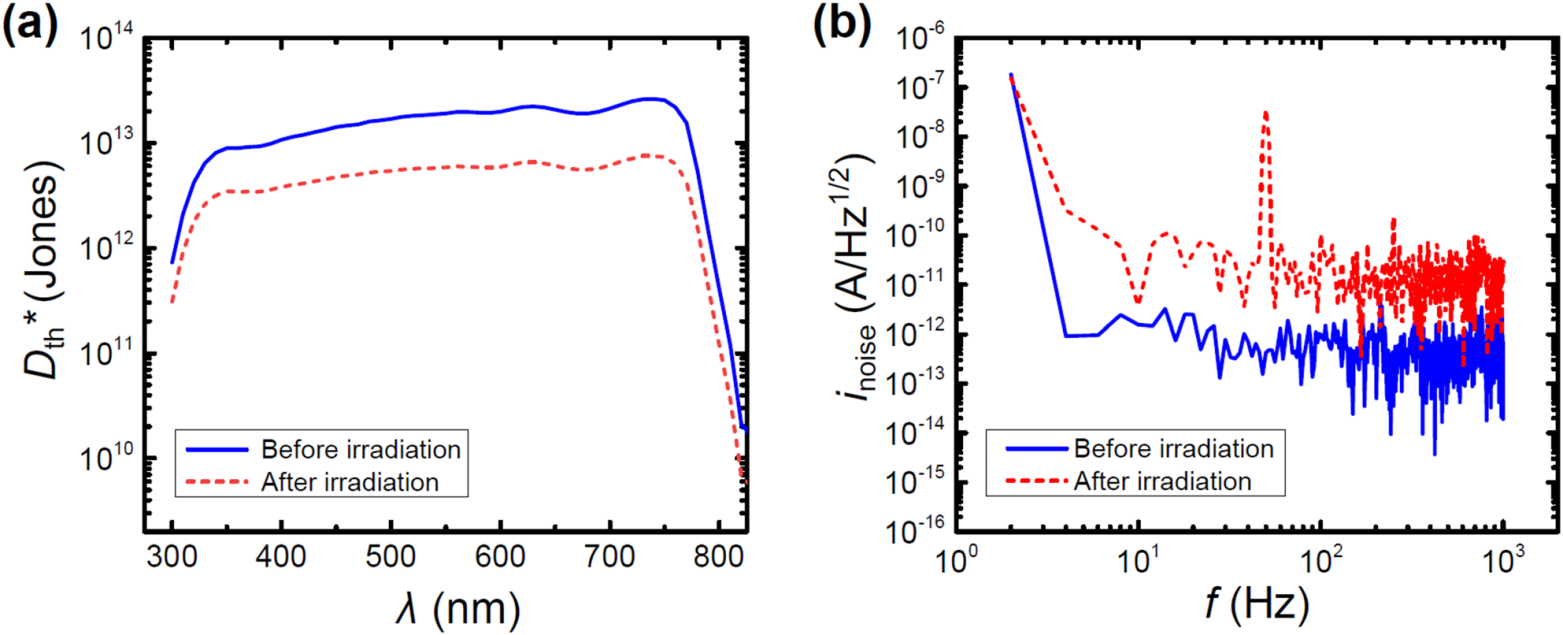
(**a**) Spectral dependence of the detectivity limited by Johnson noise and (**b**) current noise of the tested devices before and after proton irradiation.

Another important property of PDs is the characterization of their electrical noise profile, which ultimately is responsible for their detectivity. Specifically in a self-powered mode, two types of noise need to be considered: the current thermal noise *i*_thermal_, or Johnson noise, and the noise spectral density *i*_noise_. Johnson noise is linked to the load resistor, which for the tested devices is equal to the shunt resistance *R*_sh_, and independent of frequency. As a result the detectivity limited by Johnson noise *D*_th_^*^, in units of cm·Hz^1/2^/W or Jones, which evaluates the sensitivity to weak optical signals, is used as a figure-of-merit when describing PDs^35,60^:

5 $$ D_{th} \,^{*} = \frac{R\sqrt A }{{i_{thermal} }} = \frac{R\sqrt A }{{\sqrt {\frac{{2k_{B} T}}{{R_{sh} }}} }}, $$

where *R* is the responsivity, *A* is the active device area, *k*_B_ is Boltzmann’s constant, *i*_thermal_ is the thermal or Johnson noise, *T* is the absolute temperature, and *R*_sh_ is the shunt resistance. The tested devices before proton irradiation exhibit a detectivity of *D*_th_^*^ = 1 × 10^13^–3 × 10^13^ Jones over a wide spectral range of *λ* = 350–750 nm with the highest values at longer wavelengths (cf. Fig. 5). After proton irradiation, the tested devices exhibit decreased detectivity at *D*_th_^*^ = 4 × 10^12^–8 × 10^12^ Jones over the same spectral range.

Two contributing factors are responsible for the changes in *D*_th_^*^. First, the reduction in the responsivity *R* directly translates into a decreased value of* D*_th_^*^, which is specifically the case at longer wavelengths, where this reduction is most significant. Second, the increase in the dark current density, which was observed and discussed with regards to the *LDR*, plays also an important role in the changes related to the detectivity *D*_th_^*^, although its contribution is less pronounced due to the fact that it is attenuated via a square root. However, it likely is the most important factor for the reduction of *D*_th_^*^ at shorter wavelengths, since the changes in the responsivity *R* in this spectral region are less significant.

Furthermore, the noise current spectral density *i*_noise_ is another important, experimentally available characteristic that is used to properly describe PDs (cf. ESI)^19^. It can be observed that the current noise density of the tested devices increases by roughly two orders of magnitude upon proton irradiation over the entire tested frequency range (cf. Fig. 5). Furthermore, the noise current spectral density of the PDs was then utilized to determine the noise equivalent power (NEP) and specific detectivity (D^*^); the first quantifies the sensitivity of PDs with regards to the signal power resulting in a unity signal-to-noise ratio at the bandwidth of 1 Hz, whereas the second is the detectivity, if all potential sources of current noise are taken into consideration (cf. ESI). Moreover, the applicable bandwidth of a photodetector can be evaluated via its cutoff frequency *f*_−3 dB_, which is defined as the frequency at which the output of a PD is attenuated to − 3 dB (70.8%) of the original amplitude. The time dependent photoresponse of the PDs was investigated to obtain the corresponding normalized response, which in turn allows to determine the cutoff frequency *f*_−3 dB_ (cf. Figs. S7–S9). Interestingly, it was revealed that the cutoff frequency for the tested PDs before and after proton irradiation are virtually identical at *f*_−3 dB_ = 180 kHz, which likely result from the limiting RC time constant. Nonetheless, the aforementioned values indicate comparable behavior between the tested PDs before and after proton irradiation up to the RC limit. In addition, the rise times decreased after irradiation, while only small changes in the fall times of the PDs before and after proton irradiation were observed (cf. Table 3 and Fig. S10).

**Table 3:** Photodiode parameters obtained for the tested devices before and after proton irradiation.

Sample	*R*_max_ (A/W)	*D*_th_*_max_ (Jones)	*LDR*_0.0 V_ (dB)	*LDR*_−0.1 V_ (dB)	*LDR*_−0.5 V_ (dB)	*t*_rise_ (µs)	*t*_fall_ (µs)
Before irradiation	0.48	3 × 10^13^	124	124	99	7.2	3.4
After irradiation	0.35	8 × 10^12^	118	99	75	4.1	3.8

Overall, the assessed devices demonstrate promising characteristics as PDs both prior to and following proton irradiation, particularly when functioning in a self-powered mode. Although proton irradiation led to a decrease in both responsivity *R* and detectivity *D*_th_^*^, the linear dynamic range *LDR* exhibited virtually no alteration.

## Conclusion

In conclusion, MAPI-based perovskite devices fabricated through vacuum deposition were exposed to proton irradiation, resulting in changes of the electrical and optical properties. The studied solar cells demonstrated efficiencies ranking among the highest reported in the literature for this specific irradiation treatment. A thorough analysis revealed that the primary factors influencing the observed changes in performance as solar cells were a significant reduction in shunt resistance *R*_sh_ and, to a lesser extent, an increase in series resistance *R*_s_. Some changes in recombination dynamics were observed as well, although the impact on device performance is less pronounced as the aforementioned two loss processes. Furthermore, the devices maintained promising photodetector characteristics both before and after proton irradiation, especially when operated in a self-powered mode without a reverse bias. Although a decrease in responsivity *R* and detectivity *D*_th_^*^ was noted after proton irradiation, the linear dynamic range *LDR* remained largely unaffected. These results contribute valuable insights into the impact of ionizing radiation on perovskite devices manufactured via vacuum deposition and underscore their potential for applications in radiation-prone environments.

The degradation mechanism during proton irradiation likely involves the breaking of covalent bonds within the molecules of the perovskite active layer. The breaking of bonds results in the formation of localized defective states within the band gap. The location of these defective states within the band gap determines whether they act as effective recombination centres or charge traps. The presence of these centres has a detrimental effect on photovoltaic performance. The observed increase in series resistance and decrease in fill factor can be attributed to the occurrence of damage to the ETL/HTL and/or electrodes.

The present study is primarily concerned with proton-induced alterations in the physical properties of fully evaporated perovskite solar cells and photodiodes. Although we make certain assumptions based on the existing knowledge of radiation-induced degradation mechanisms in perovskite optoelectronic devices, further comprehensive and multifaceted material characterization of all functional layers that make up the device stack is required to unravel the exact degradation mechanisms in proton-irradiated perovskite optoelectronic devices.

### Supplementary Information

Supplementary Information.

## Data Availability

The materials and data that support the findings of this study are available from the corresponding authors on request.
