# Supplementary material for: Characterization of fully-evaporated perovskite solar cells and photodetectors under high-intensity pulsed proton irradiation
Source: Sci Rep. 2024 Jul 20;14:16735. doi: 10.1038/s41598-024-67541-5 (PMC11271285; doi:10.1038/s41598-024-67541-5)
Supplement: Supplementary file 1 — Supplementary Information. [file 41598_2024_67541_MOESM1_ESM.docx]

Supplementary Information

**Characterization of Fully-Evaporated Perovskite Solar Cells and Photodetectors under High-Intensity Pulsed Proton Irradiation**

**Hryhorii P. Parkhomenko^1^, Andriy I. Mostovyi^1^, Marat Kaikanov^1^, Jessica Strey^2^, Mircea C. Turcu^2^, Marvin Diederich^2^, Sascha J. Wolter^2^, Verena Steckenreiter^2^, Joachim Vollbrecht^2,^*, and Viktor V. Brus^1,^***

^1^Department of Physics, School of Sciences and Humanities, Nazarbayev University, Astana 010000, Republic of Kazakhstan

^2^Department of Photovoltaics, Institute for Solar Energy Research Hamelin, Emmerthal 31860, Germany

*******Corresponding authors: E-mail: vvbrus@gmail.com, vollbrecht@isfh.de

**MATERIALS**

The chemical compounds PbI_2_, C60, bathocuproine (BCP) – all from Sigma-Aldrich – and 2,2′,7,7′-tetrakis(di-p-tolylamino)spiro-9,9′-bifluorene (spiroTTB, Lumtec) were used without further purification. MAI was synthesized by stirring methyl amine solution (40%, Sigma-Aldrich) and aqueous hydrogen iodide (57%, Thermo Fischer) for 2 h at 0 °C. A rotary evaporator was employed for water extraction and the remaining primary MAI was rinsed for at least three times in diethyl ether.

**DEVICE FABRICATION**

First, ITO-coated glass substrates with a square geometry (2.5 x 2.5 cm^2^) were cleaned in an ultrasonic bath in three consecutive steps using mucasol, isopropanol and acetone. After the mucasol step they were then rinsed with water to remove any remaining soap residues. Then, the substrates were subjected to a UV/ozone treatment. Subsequently, the substrates were placed in a substrate holder, where the temperature was kept at 25°C during the following deposition processes. Then, 10 nm spiroTTB were thermally evaporated (ν = 0.25 Å/s) as hole transporting layer (HTL) in a lab-type evaporation chamber (MiniSPECTROS, Kurt J. Lesker Company) at a processing pressure of *p* < 3 x 10^-6^ mbar. Subsequently, an active layer of 500 nm thick co-evaporated MAPI was deposited. The PbI_2_ crucible was heated to 200°C. Reaching that temperature, the MAI crucible was heated to 100°C while the PbI_2_ crucible was further heated to 300°C. After opening the crucible shutters and reaching a PbI_2_ deposition rate of 1.08 Å/s as well as a MAI crucible temperature of 118°C, the co-evaporation process started with the subsequent opening of the substrate shutter. To ensure a laterally homogeneous deposition, the substrate holder was rotated at 20 rpm. In the same chamber, 23 nm C60 (ν = 0.25 Å/s) directly followed by 8 nm BCP (ν = 0.25 Å/s) were evaporated to form the electron transporting layer (ETL) of the devices. The evaporation chamber was directly connected to an N_2_-filled glovebox and the devices were then transferred under N_2_-atmosphere to another evaporation chamber (Nano36, Kurt J. Lesker Company) for electrode deposition. The devices were finished by the evaporation of 100 nm Cu using a ramped evaporation program (10 nm at ν_1_ = 0.2 Å/s, 20 nm at ν_2_ = 1.0 Å/s, 40 nm at ν_3_ = 2.0 Å/s, 30 nm at ν_4_ = 4.0 Å/s). The overlap of the Cu-electrodes and the ITO-anodes defined the active area of the devices (*A* = 12 mm^2^).

**PROTON IRRADIATION**

The specimens were subjected to irradiation at the INURA pulsed high-current proton accelerator. It is noteworthy that our experiment involved the exposure of an irradiated device, accompanied by reference devices. These reference devices, although exempt from irradiation, were consistently positioned in close proximity to the irradiated samples and subjected to identical atmospheric conditions. Notably, the reference devices exhibited no discernible alterations, indicative of the minimal impact of the ambient atmosphere on the intrinsic characteristics of the devices. Throughout the irradiation process, the samples were situated within a vacuum chamber on a rotating table, positioned at a distance of 35 cm from the diode. The chamber was evacuated to a pressure of 5 x 10^-2^ mbar. Preceding the irradiation of the specimens, the proton beam parameters underwent continuous monitoring through the utilization of a collimated Faraday cup featuring a magnetic cut-off. A minimum of ten controlled proton beam impulses were applied to the Faraday cup to ensure the stability of the beam parameters. The amplitude of the accelerating voltage was established at 140 kV, and the peak proton beam current density reached approximately 4.5 A/cm^2^. The proton beam accumulated fluence was computed from the proton beam current density oscillogram by integrating it over time, resulting in a cumulative exposure of 2·10^12^ p/cm^2^. This substantial dose was administered within a brief temporal window of 150 ns during the proton beam current impulse. The devices underwent irradiation with three pulses, culminating in a total fluence of 6·10^12^ p/cm^2^. It is imperative to underscore that the proton irradiation conditions employed in this study were exceptionally rigorous, surpassing the genuine conditions associated with intense cosmic radiation.

**DEVICE CHARACTERIZATION**

The initial characterization of the devices was performed in an inert, N_2_-filled glovebox. The *J*-*V*-characteristics of the tested devices were obtained via a custom-made setup employing a W-lamp in combination with a calibrated reference photodiode, resulting in adjusted illumination conditions (intensity = 100 mW/cm^2^). The semiconductor Device Analyzer (B1500A, Keysight) was employed in conjunction with an AAA solar simulator (Oriel, Newport) under standard conditions (intensity = 100 mW/cm^2^, 1.5 AM) to measure the performance of the devices. An external quantum efficiency measurement system (ORIEL IQE 200, Newport) facilitated the assessment of *EQE* spectra at varying reverse biases. Open-Circuit Voltage Decay (OCVD) measurements were conducted under 100 mW/cm^2^ light exposure using a rapid-switching white light-emitting diode regulated by a fast-switching driver and a square wave function generator (SDG6032X, Siglent). The solar cells were linked to a digital oscilloscope (SDS5032X, Siglent) through a high impedance buffer (5 GΩ), featuring 200 MHz bandwidths, within a Faraday cage to ensure appropriate grounding. A Solartron SI1260 analyzer was employed to gauge frequency-dependent impedance spectra (10^1^ - 10^7^ Hz) under 1 sun and in dark conditions. This analysis involved a small AC disturbance of 40 mV, initially applying a negative sample bias and incrementally increasing the bias up to *V*_OC_. The determination of the linear dynamic range entailed acquiring *J*-*V*-curves across a wide spectrum of intensities utilizing distinct optical density filters (NEK01S, Thorlabs). To ascertain response time and cutoff frequency, a high-speed blue LED coupled with a 200 MHz bandwidth driver, controlled by a 350 MHz square wave function generator (SDG6032X, Siglent), alongside a 350 MHz-bandwidth oscilloscope (SDS5032X, Siglent), was utilized. For the assessment of current noise in the photodiodes, a battery-powered low-noise current amplifier (SR570, Stanford Research Systems) combined with the SDS5032X oscilloscope operating in the Fast Fourier Transform (FFT) mode was employed within a Faraday cage.


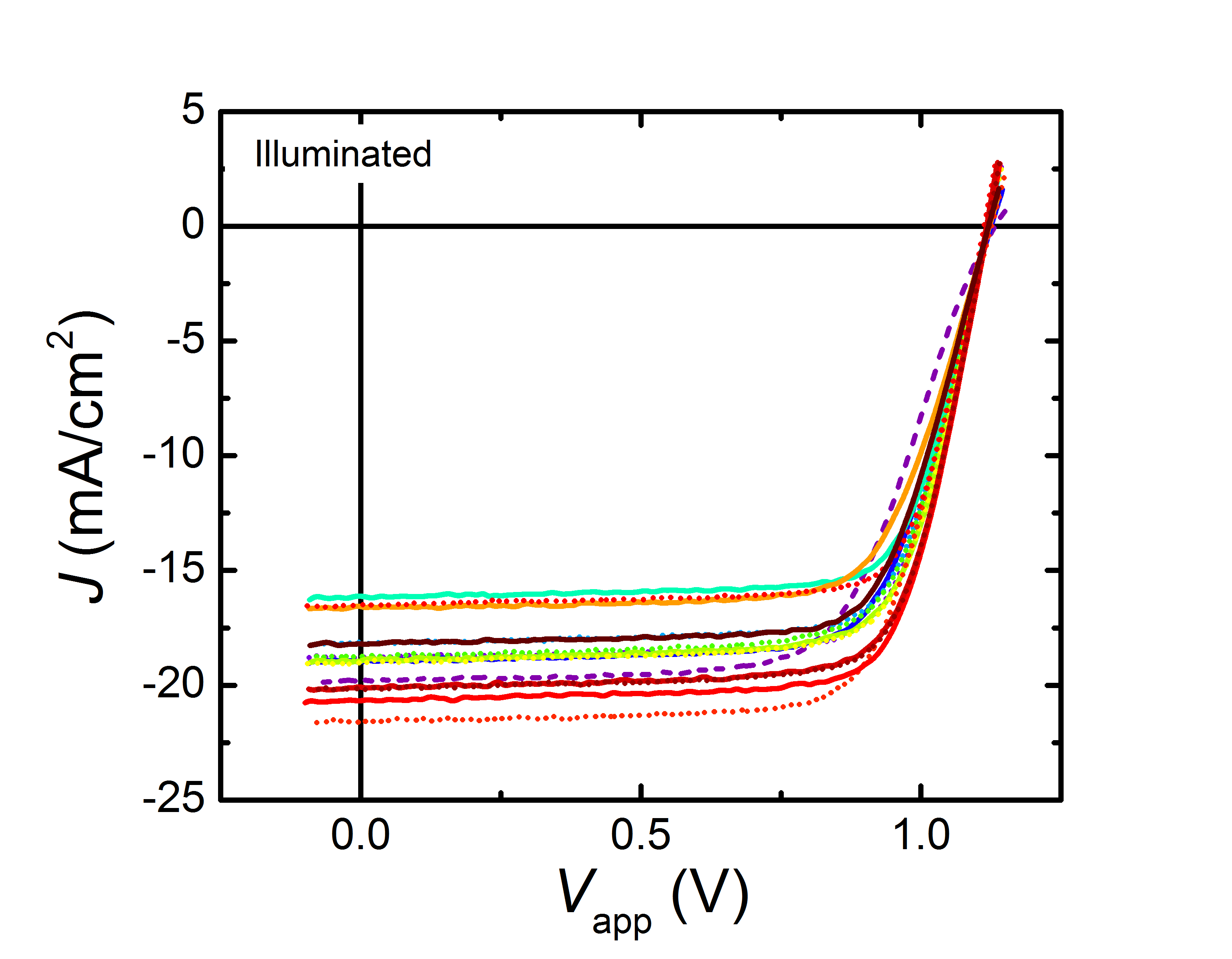


**Figure S1.** Initial *J*-*V*-characteristics of the tested batch of MAPI devices under 1 sun illumination.

**DETERMINATION OF CHARGE CARRIER DENSITY**

The underlying principle to determine the charge carrier density *n* is based on the link between the chemical capacitance *C*_µ_ and *n*:

$C_{\mu}=qAL\frac{dn}{dV}$, (S1)

where *q* is the elementary charge, *L* is the active layer thickness, *A* is the device area, and *V* is the voltage. Rearrangement of Eq. S1 leads to:

$n=\frac{1}{qAL}\int C_{\mu}dV$. (S2)

The determination of *C*_µ_ was carried out via impedance spectroscopy (IS) measurements. However, a direct measurement of *C*_µ_ is not possible and several corrections and assumptions are usually required to obtain *C*_µ_. While this approach has been used successfully in organic solar cells numerous times, perovskite devices are challenging to analyze due to the high dielectric constant of perovskite layers compared to its organic transport layers (ε_r,pero_≈ 30 – 40 and ε_r,organic_ ≈ 3, respectively), since a relationship of capacities in series is assumed. As a result, great care has to be taken to ensure that the capacities of the active layers are not mistaken for the capacities resulting from the organic transport layers. In this study, the difference between the measured barrier capacitances *C*_b_ in the dark and under illumination of the identical devices was assumed to be approximately the chemical capacitance *C*_µ_ of the perovskite layer, leading to the following relationship:

$n=\frac{1}{qAL}\int_{0}^{V_{\mathrm{OC}}} \left( C_{b,light}-C_{b,dark} \right)dV$. (S3)

This approach, which resembles method V in Ref. [47] of the main manuscript, also sidesteps the requirements for corrections related to the geometric capacitance *C*_g_ and capacitances related to the transport layers, since these parameters should not significantly change upon illumination. The measured capacitance *C*_b_ at a frequency of *f*= 1389 Hz was chosen, since the unwanted influence of parasitic effects due to trap states or ion migration that occur usually at lower frequencies and effects due to parasitic inductance normally occurring at higher frequencies, can be minimized (cf. Figure S1 and S2).


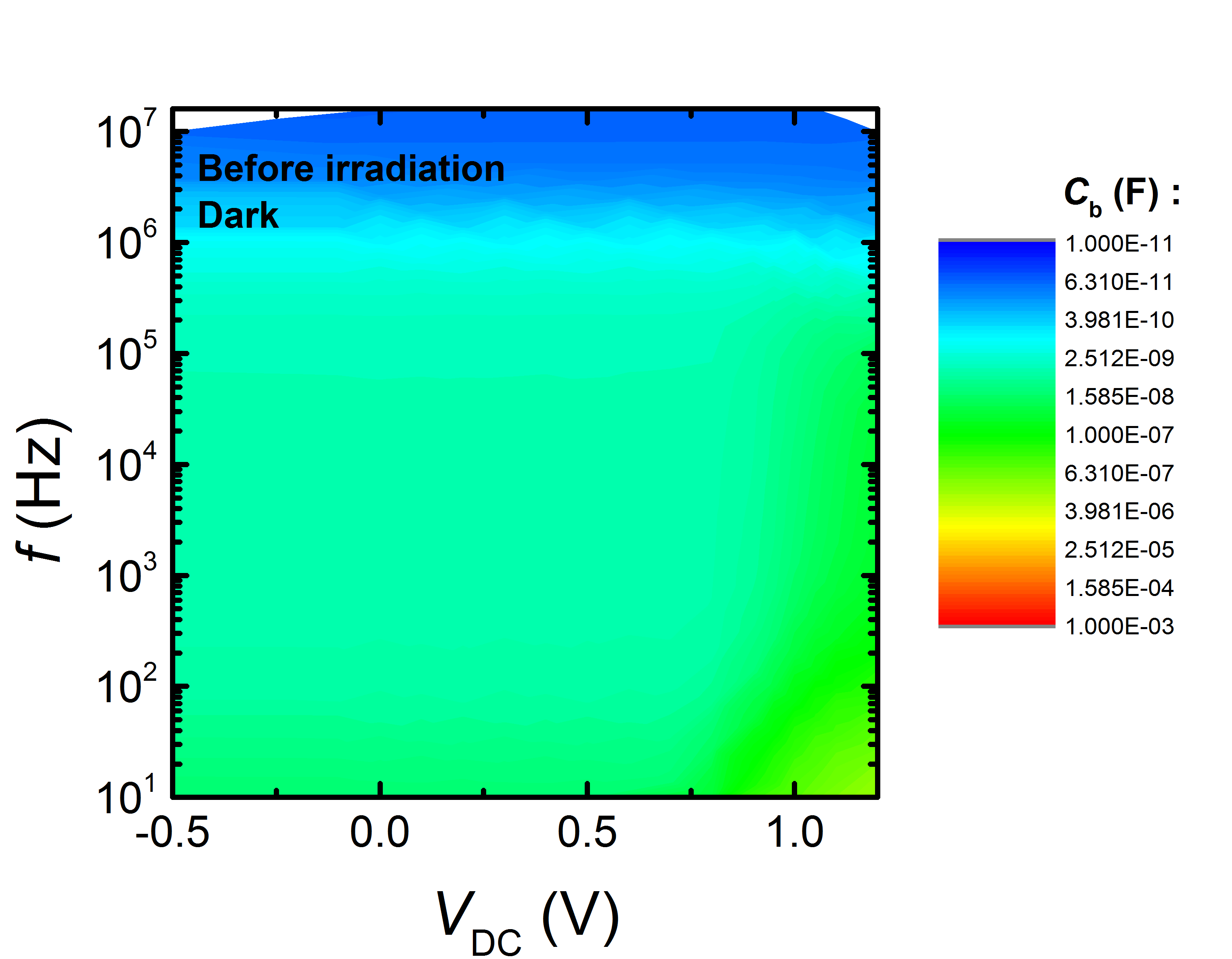

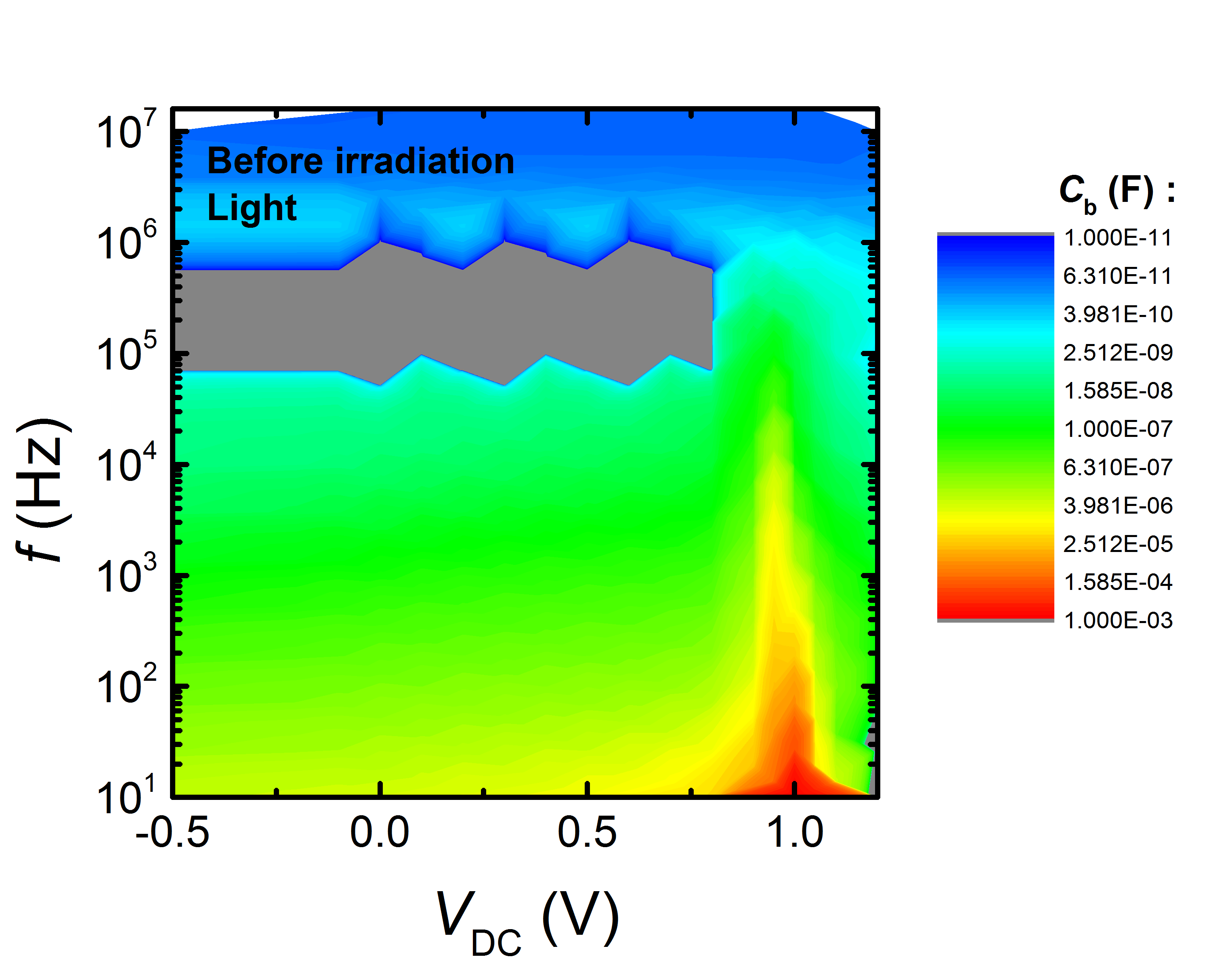

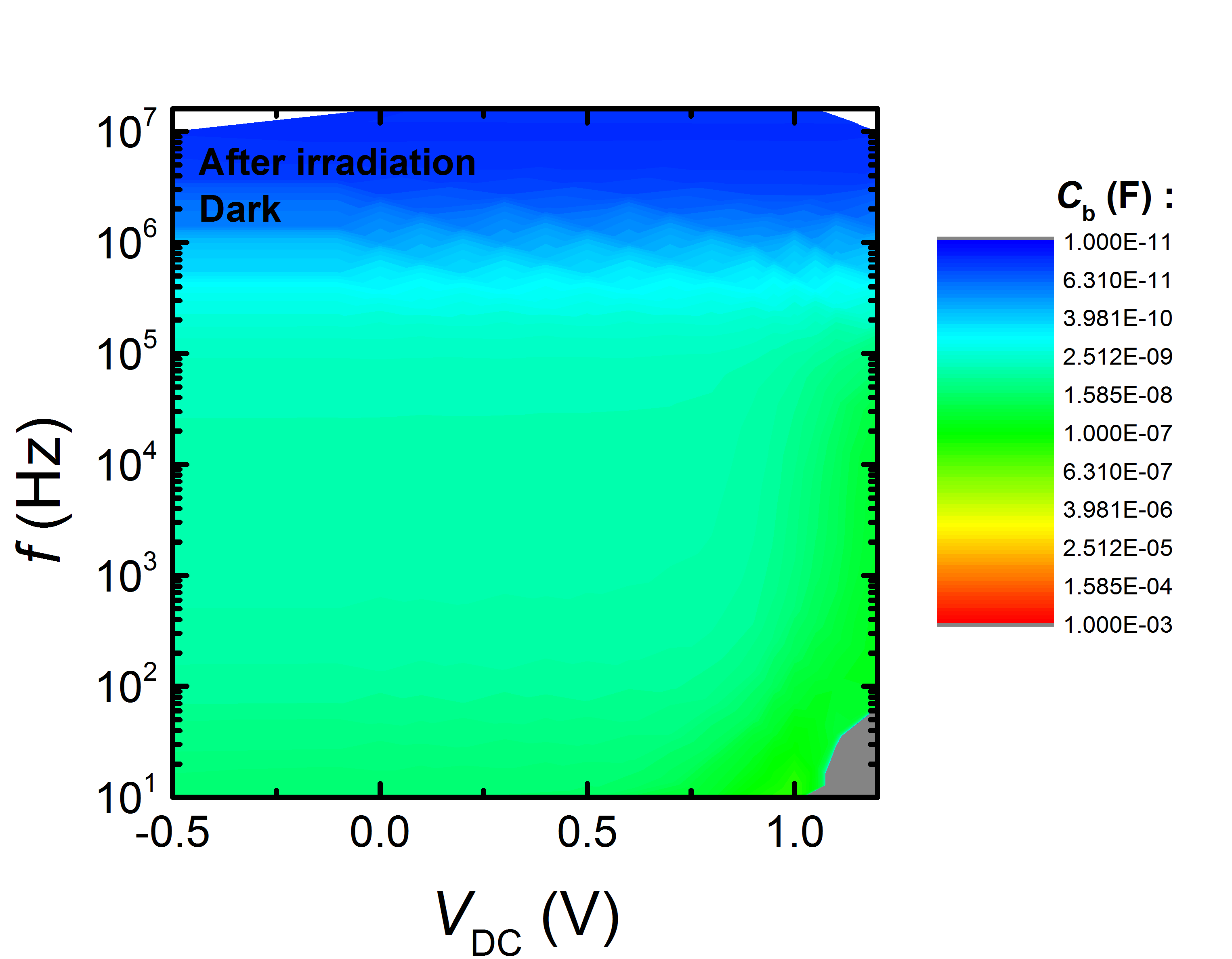

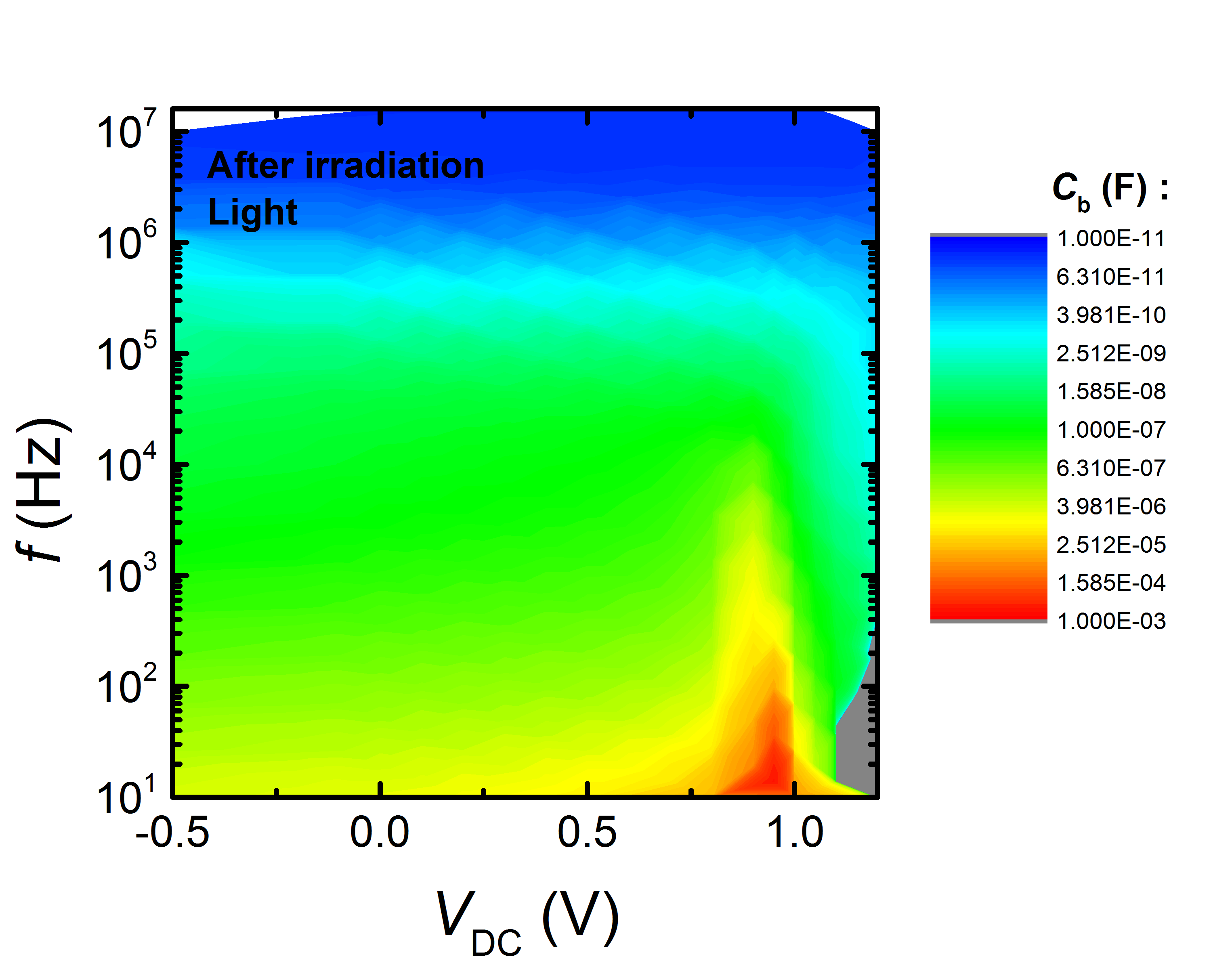
 **Figure S2.** Barrier capacitance *C*_b_ in dependence of the applied DC-voltage *V*_DC_ and the frequency *f* in the dark and under illumination of 1 sun for the studied cell before and after proton irradiation. Areas with undefined values (e.g. *C*_b_ < 0) are denoted in gray.


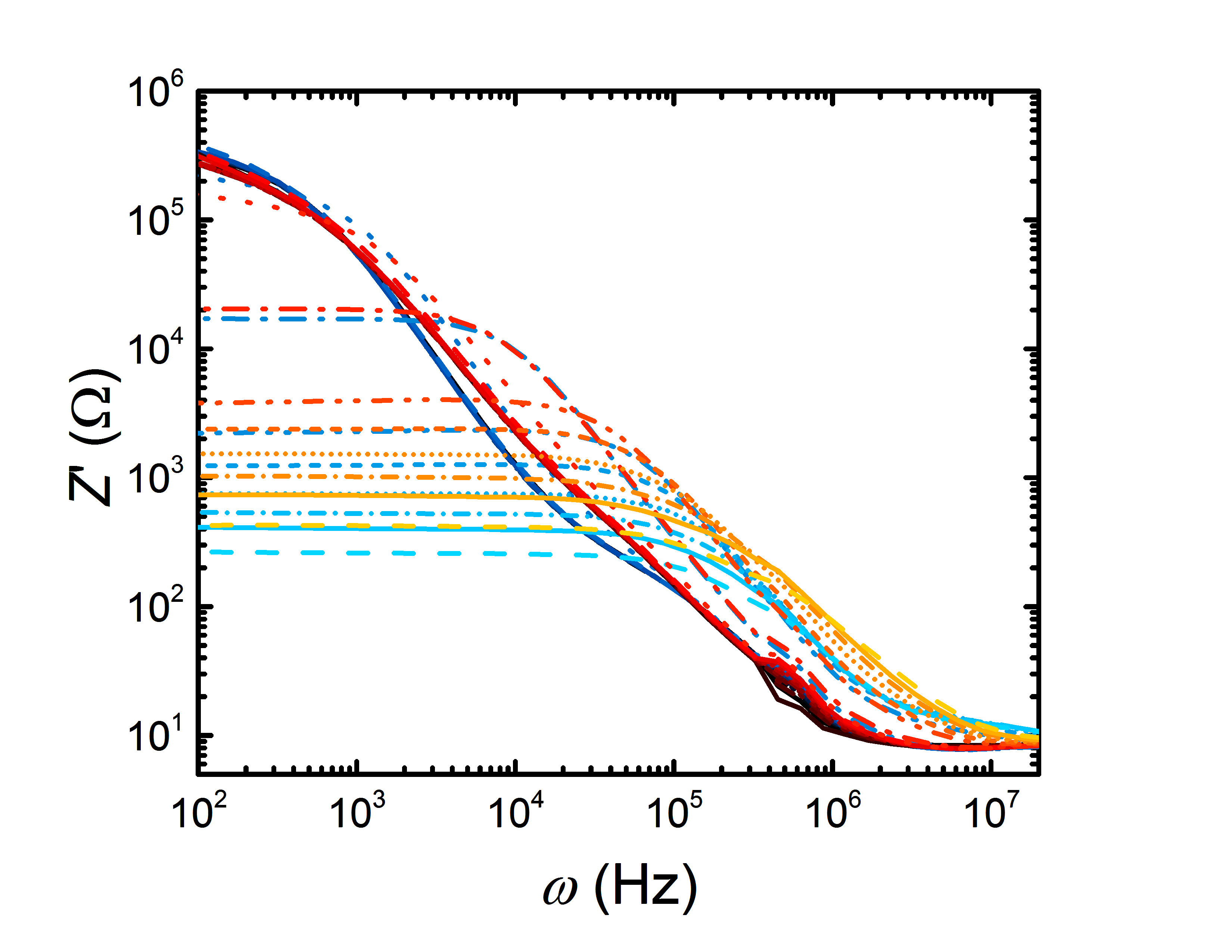

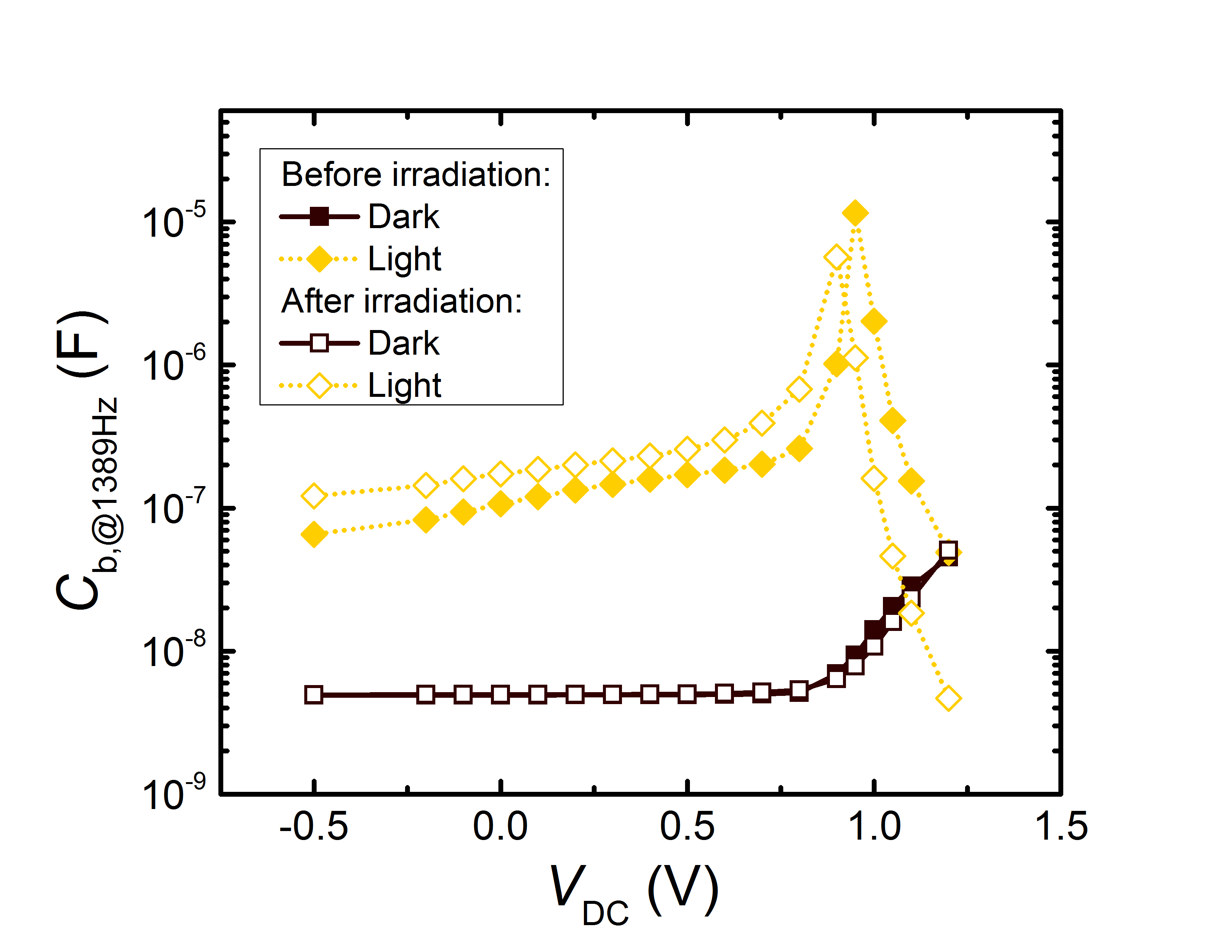

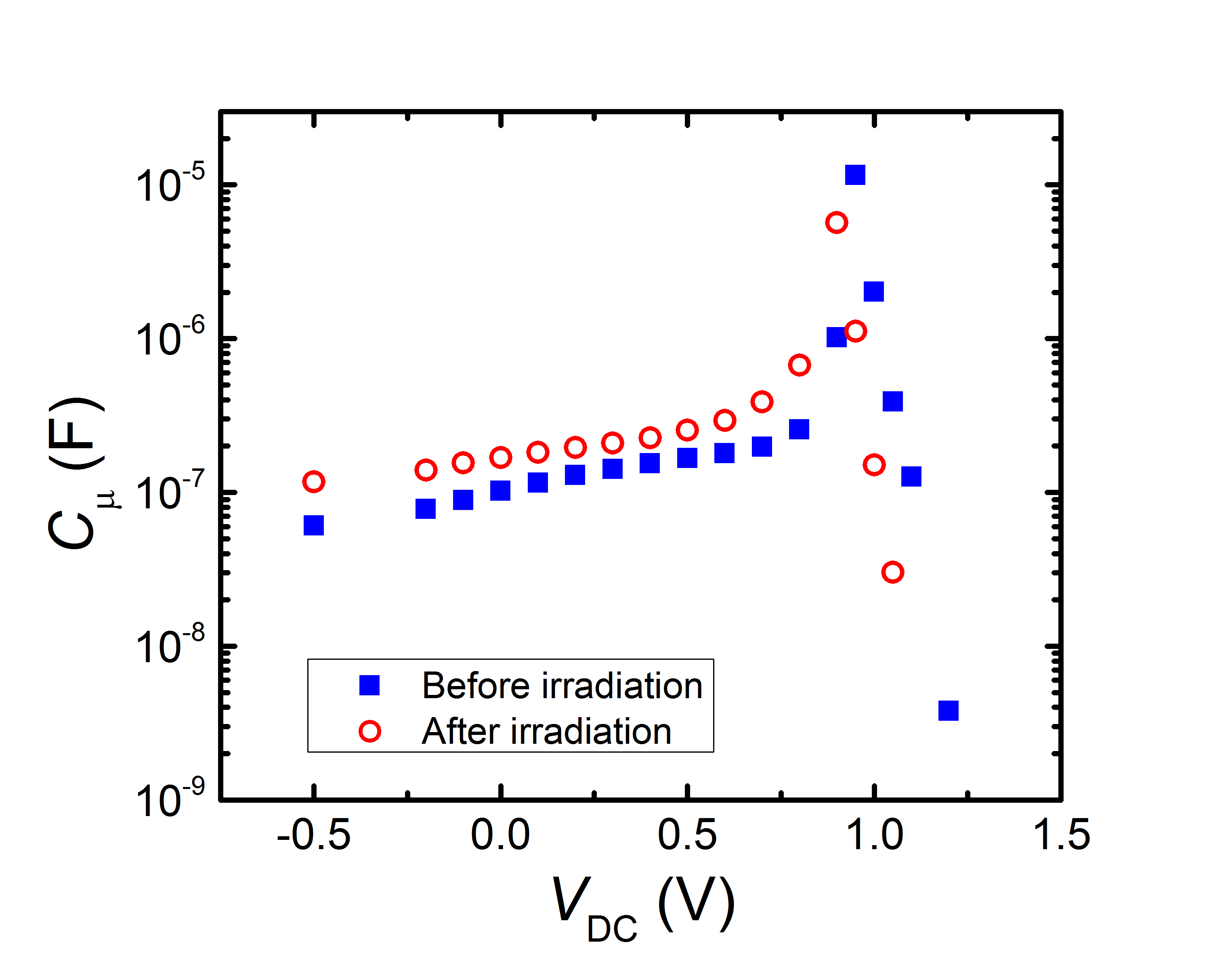

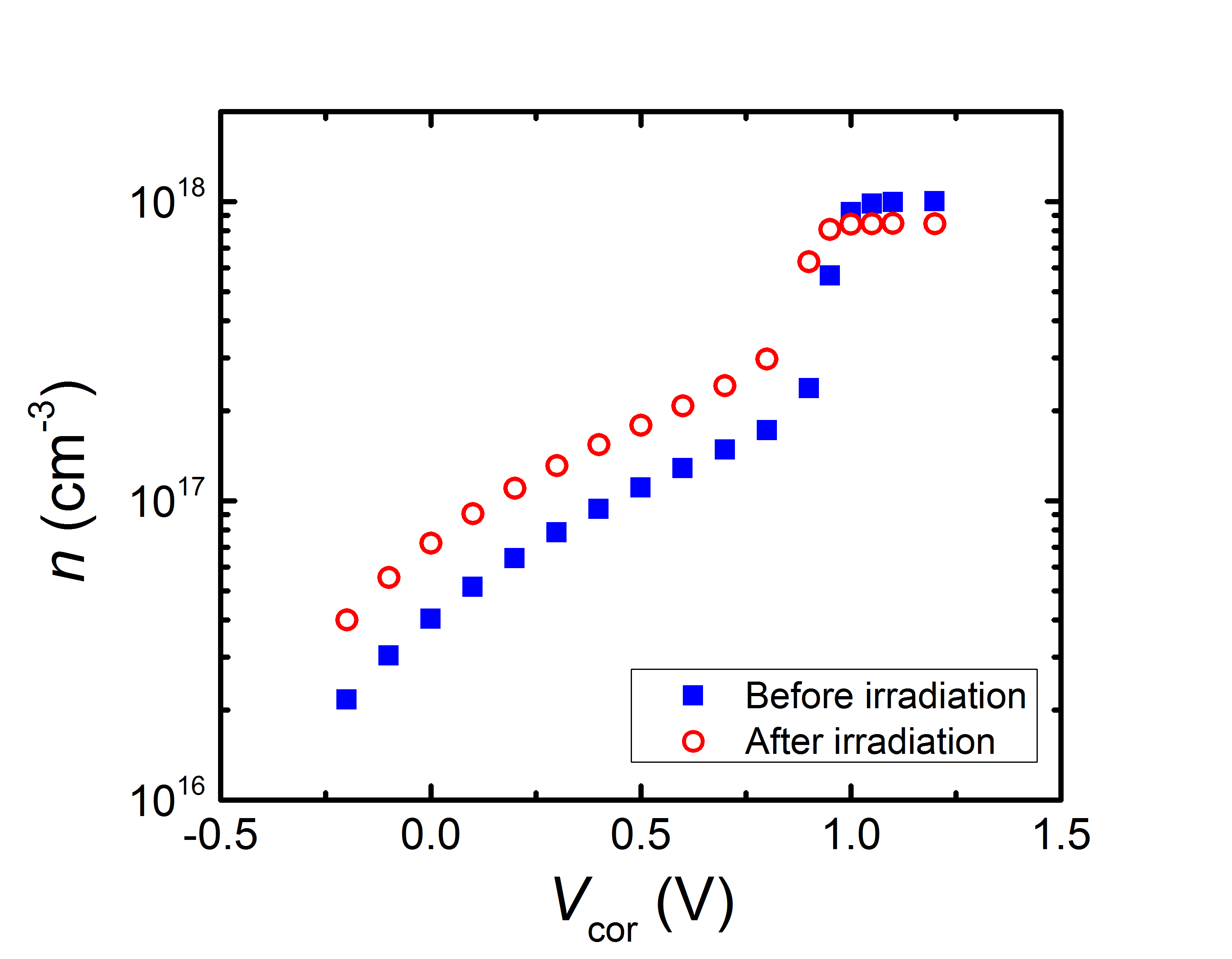


**Figure S3.** Angular frequency *ω* dependent dark impedance *Z*' of the device before (blue) and after (red) irradiation at various applied voltages *V*_DC_. Voltage-dependent barrier capacitance *C*_b_ at *f* = 1389 Hz in the dark and under 1 sun illumination before and after irradiation. Voltage-dependent chemical capacitance *C*_µ_ of the device before and after irradiation. Charge carrier density dependence on corrected voltage (*V*_cor_ = *V*_DC_-*J⸱R*_s_) before and after proton irradiation.

**QUANTITATIVE ANALYSIS OF RECOMBINATION DYNAMICS**

The determination of the time-dependent charge carrier density *n*(*t*) is an important prerequisite to obtain quantitative insights with regard to the recombination rates *U* and recombination coefficients *k*. The most common approach is to use a secondary technique other than OCVD to obtain time-dependent values for the charge carrier density. In this study, the determination of the charge carrier density, specifically under open-circuit conditions that was described above, allows the necessary transformation of the time-dependent open-circuit voltage *V*_OC_(*t*) to the time-dependent charge carrier density *n*_OC_(*t*) via the following relationship:

$\boldsymbol{n}_{\mathbf{OC}}\left( \boldsymbol{t} \right)\boldsymbol{=}\boldsymbol{n}_{\mathbf{i}}\boldsymbol{\cdot}\mathbf{exp}\left\{ \frac{\boldsymbol{q}\boldsymbol{V}_{\mathbf{OC}}\left( \boldsymbol{t} \right)}{\boldsymbol{2}\boldsymbol{k}_{\mathbf{B}}\boldsymbol{T}} \right\}$, (S4)

where *n*_i_ is the intrinsic charge carrier density, *q* is the elementary charge, *k*_B_ is Boltzmann’s constant, and *T* is the absolute temperature. The intrinsic charge carrier density *n*_i_ can be calculated, since *V*_OC_(*t*= 0) at 1 sun is known from OCVD and *n*_OC_(*t* = 0) at 1 sun is known from IS. With *n*_i_ now known, all *V*_OC_(*t*) values can be transformed into *n*_OC_(*t*) values according to Eq. S4. Furthermore, the total recombination rate *U*_tot_ and the recombination lifetime *τ* are linked via:

$\boldsymbol{U}_{\mathbf{tot}}\left( \boldsymbol{t} \right)\boldsymbol{=}\frac{\boldsymbol{dn}\left( \boldsymbol{t} \right)}{\boldsymbol{dt}}\boldsymbol{=-}\frac{\boldsymbol{n}\left( \boldsymbol{t} \right)}{\boldsymbol{\tau}\left( \boldsymbol{t} \right)}$. (S5)

Hence, it is possible to calculate the recombination lifetime *τ* using the now available time-dependent charge carrier density *n*_OC_(*t*) (cf. Figure S3).


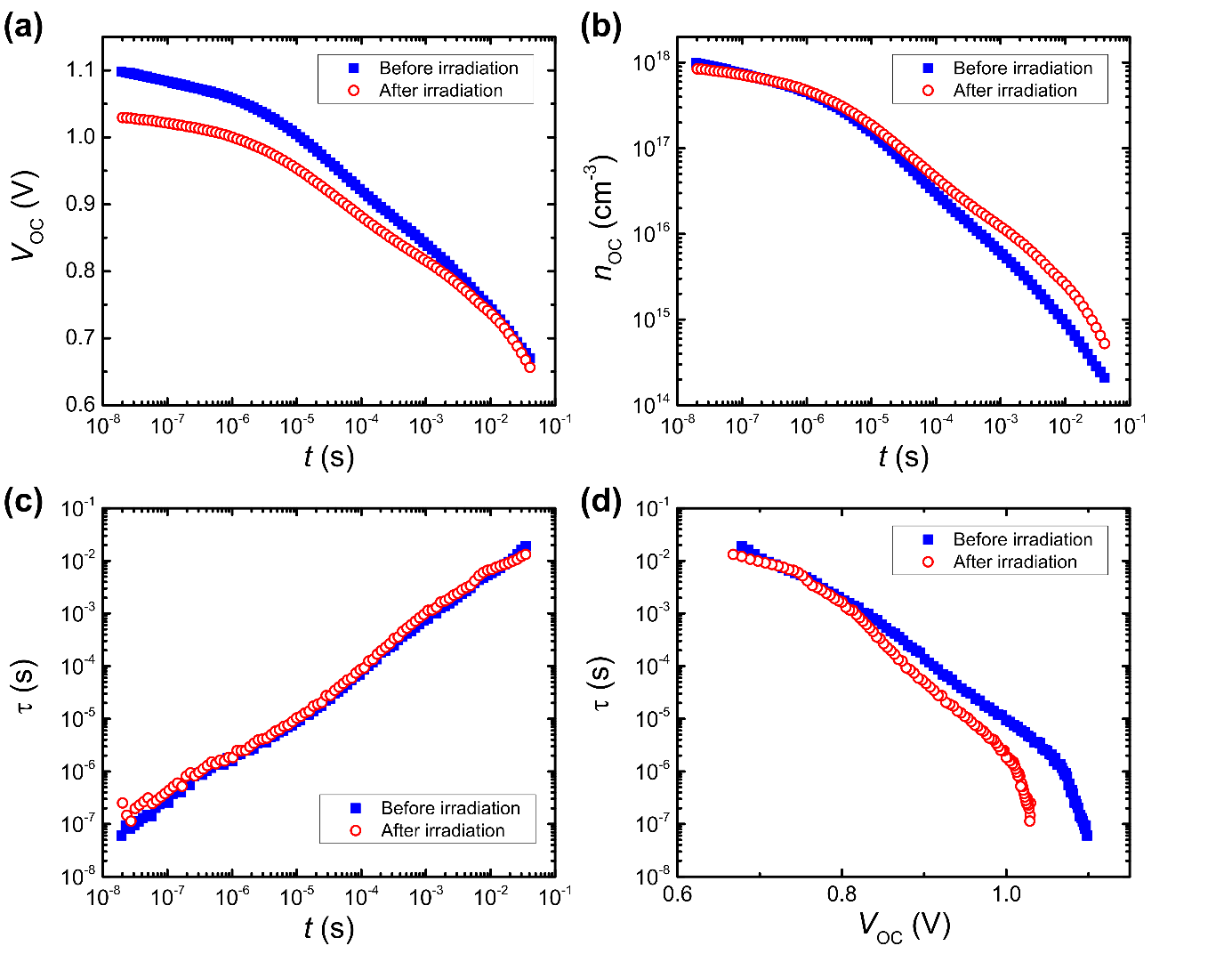


**Figure S4.** Transient open-circuit voltage decay characteristics of the tested devices before and after proton irradiation: (a) Time dependent open-circuit voltage, (b) time dependent open-circuit charge carrier density, (c) time dependent effective recombination lifetime, and (d) open-circuit voltage dependent effective recombination lifetime.

First order recombination processes, such as trap-assisted recombination in the bulk, are described by the following relationship:

$\boldsymbol{U}_{\boldsymbol{1}}\left( \boldsymbol{t} \right)\boldsymbol{=}\frac{\mathbf{d}\boldsymbol{n}\left( \boldsymbol{t} \right)}{\mathbf{d}\boldsymbol{t}}\boldsymbol{=-}\boldsymbol{k}_{\boldsymbol{1}}\boldsymbol{\cdot}\boldsymbol{n}\left( \boldsymbol{t} \right)$, (S6)

where *n*(*t*) is the transient charge carrier density, *U*_1_(*t*) is the recombination rate and *k*_1_ is the recombination coefficient of first order processes. Second order recombination processes, such as band-to-band recombination, are described as follows:

$\boldsymbol{U}_{\boldsymbol{2}}\left( \boldsymbol{t} \right)\boldsymbol{=}\frac{\mathbf{d}\boldsymbol{n}\left( \boldsymbol{t} \right)}{\mathbf{d}\boldsymbol{t}}\boldsymbol{=-}\boldsymbol{k}_{\boldsymbol{2}}\boldsymbol{\cdot}{\boldsymbol{n}\left( \boldsymbol{t} \right)}^{\boldsymbol{2}}$, (S7)

where *U*_2_(*t*) is the recombination rate and *k*_2_ is the recombination coefficient of second order processes. Third order and pseudo-third order recombination processes, such as Auger recombination and surface trap-assisted recombination, can be expressed by:

$\boldsymbol{U}_{\boldsymbol{3}}\left( \boldsymbol{t} \right)\boldsymbol{=}\frac{\mathbf{d}\boldsymbol{n}\left( \boldsymbol{t} \right)}{\mathbf{d}\boldsymbol{t}}\boldsymbol{=-}\boldsymbol{k}_{\boldsymbol{3}}\boldsymbol{\cdot}{\boldsymbol{n}\left( \boldsymbol{t} \right)}^{\boldsymbol{3}}$*,* (S8)

where *U*_3_(*t*) is the recombination rate and *k*_3_ is the recombination coefficient of third and pseudo-third order processes.

Under realistic conditions, solar cells tend to exhibit combinations of the three aforementioned recombination mechanisms and it is thus assumed that a superposition of these three processes is a good approximation for the total recombination rate *U*_tot_(*t*):

$\boldsymbol{U}_{\mathbf{tot}}\left( \boldsymbol{t} \right)\boldsymbol{=}\boldsymbol{U}_{\boldsymbol{1}}\left( \boldsymbol{t} \right)\boldsymbol{+}\boldsymbol{U}_{\boldsymbol{2}}\left( \boldsymbol{t} \right) {\boldsymbol{+}\boldsymbol{U}}_{\boldsymbol{3}}\left( \boldsymbol{t} \right)$. (S9)

Once the recombination lifetime *τ* is plotted against the transient charge carrier density *n*_OC_(*t*), it is possible to approximate the experimental results via a fit that includes the three recombination coefficients *k*_1_, *k*_2_, and *k*_3_ as fitting parameters:

$\boldsymbol{\tau=}\frac{\boldsymbol{n}}{\left( \boldsymbol{k}_{\boldsymbol{1}}\boldsymbol{n+}\boldsymbol{k}_{\boldsymbol{2}}\boldsymbol{n}^{\boldsymbol{2}}\boldsymbol{+}\boldsymbol{k}_{\boldsymbol{3}}\boldsymbol{n}^{\boldsymbol{3}} \right)}$. (S10)


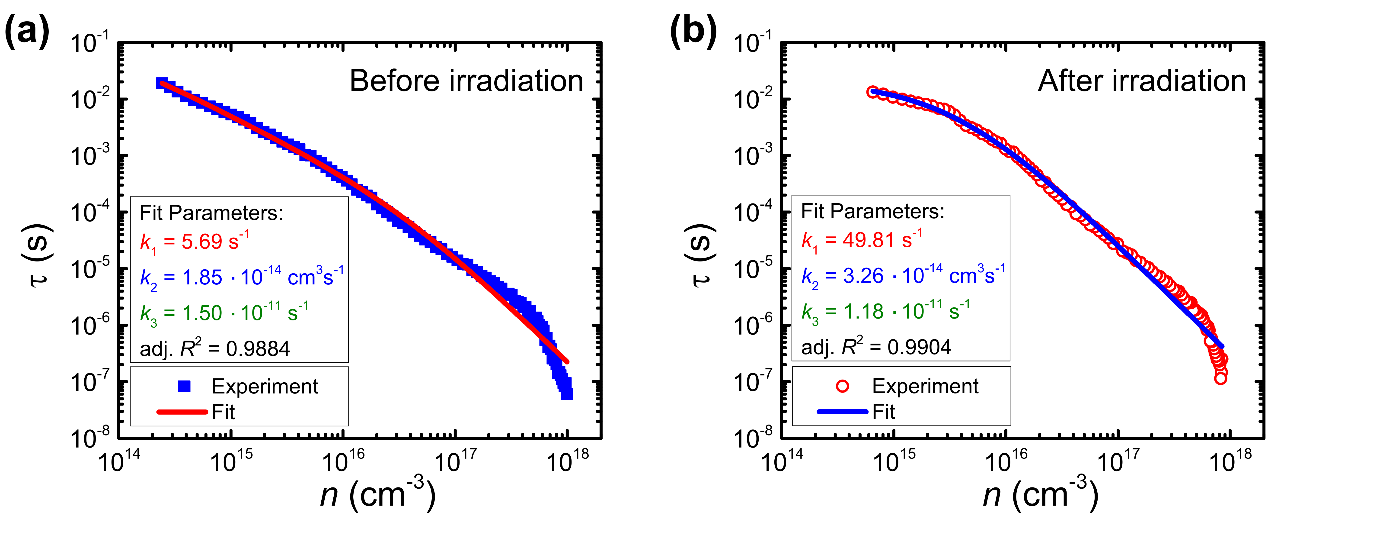


**Figure S5.** Experimentally determined effective recombination lifetimes (symbols) and analytical fits (solid lines) as a function of the charge carrier density for the tested devices (a) before and (b) after proton irradiation.

**CHARACTERIZATION OF PHOTODETECTOR PROPERTIES**

The noise spectral density, otherwise also described as current noise density, *i*_noise_ is the net current resulting from all types of noise mechanisms:

$i_{\mathrm{noise}}=\sqrt{i_{\mathrm{thermal}}^{2}+i_{\mathrm{shot}}^{2}+i_{1/f}^{2}\cdots}$, (S11)

Where *i*_thermal_ is the thermal noise, *i*_shot_ is the shot noise, and *i*_1/_*_f_* is the flicker noise. There are other potential sources of noise, such as trapping and detrapping. Experimentally, the following relationship was used to determine the noise current spectral density:

$i_{\mathrm{noise}}=G\sqrt{\frac{R_{L}P_{\mathrm{out}}\left( f \right)}{BW}}$, (S12)

Where *G* is the transimpedance gain of the used amplifier in A/V, *R*_L_ = 50 Ω as the input resistance of the oscilloscope, *P*_out_( *f*) is the difference between measured power spectra of the tested PD and the background noise of the measurement setup, and *BW* is the bandwidth. Moreover, the specific detectivity *D*^*^ of the tested PDs based on the experimentally determined *i*_noise_ is defined via:

$D^{*}=\frac{R\sqrt{A}}{i_{\mathrm{noise}}},$ (S13)

Where *R* is the responsivity, *A* is the active area of the tested PDs, and *i*_noise_ is the noise spectral density as described above in Eq. S12. See Figure S6 for the wavelength and and frequency dependent specific detectivity *D*^*^. The noise equivalent power (NEP) is another important metric used to quantify the sensitivity of PDs with regards to the signal power that results in the unity signal-to-noise ratio at the bandwidth of 1 Hz. It can be determined by using the following equation:

$\mathrm{NEP}=\frac{i_{\mathrm{noise}}}{R}$, (S14)

where *R* stands for the responsivity of the photodiode, and *i*_noise_ is the noise current spectral density. The wavelength and frequency dependent NEP is shown in Figure S6.


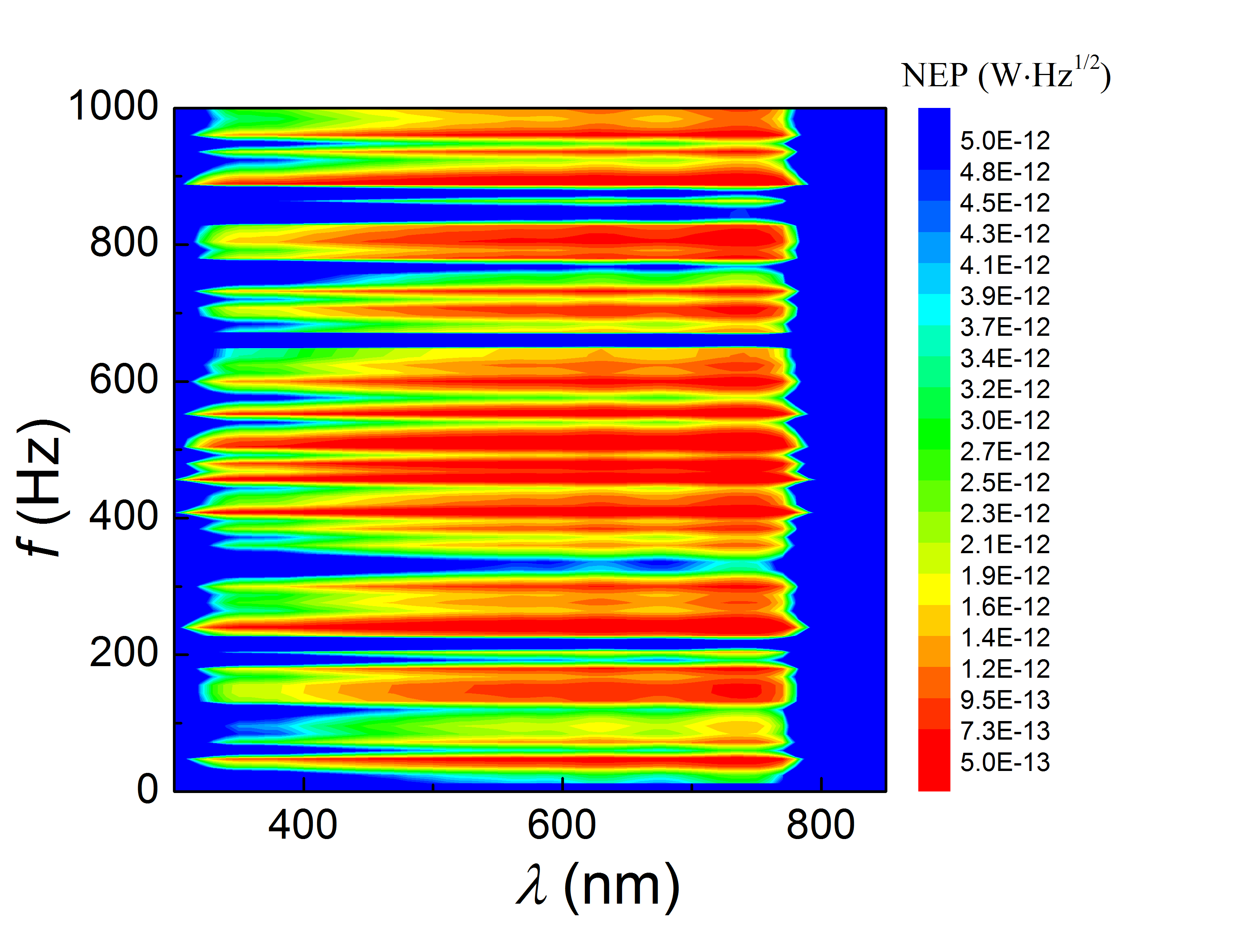

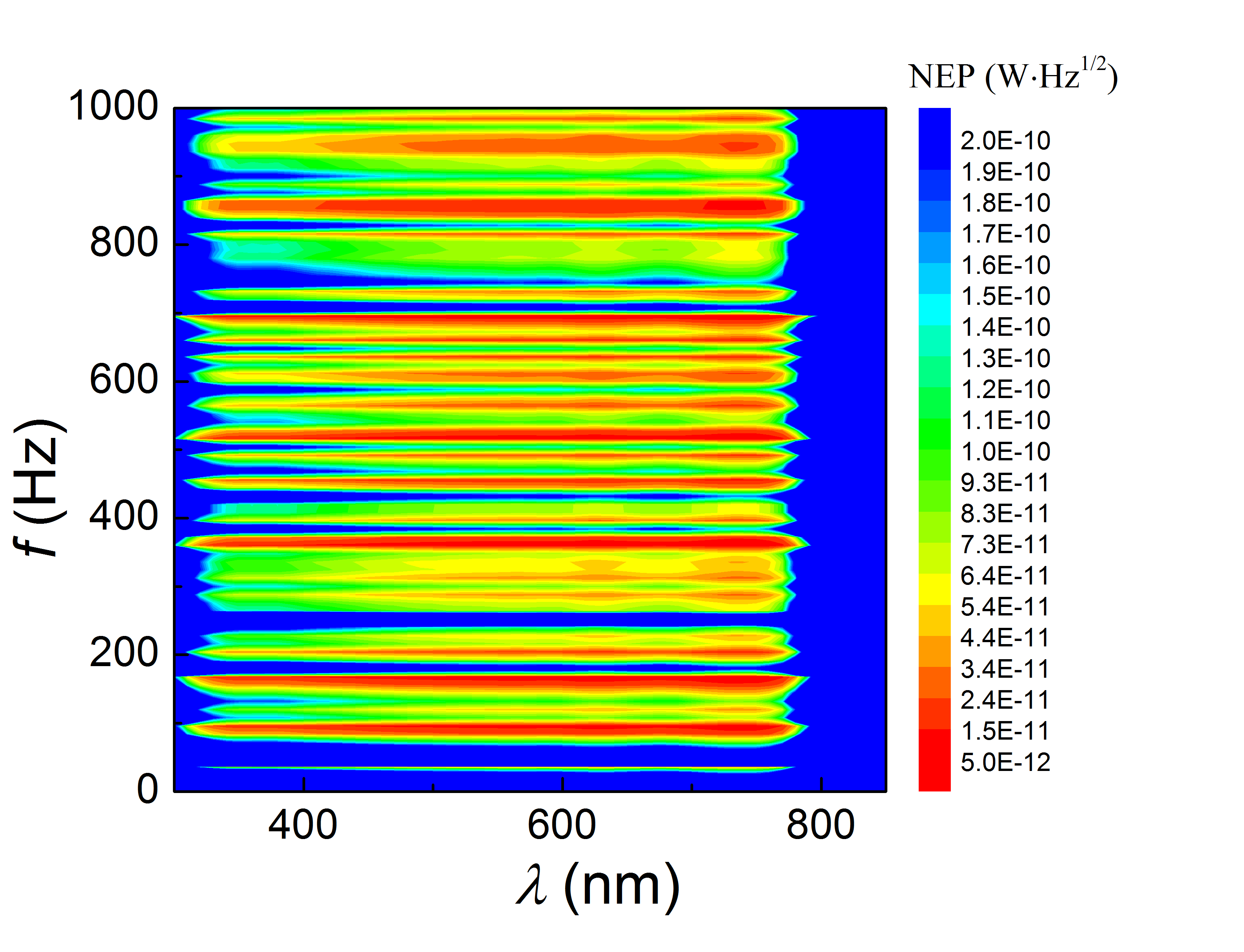

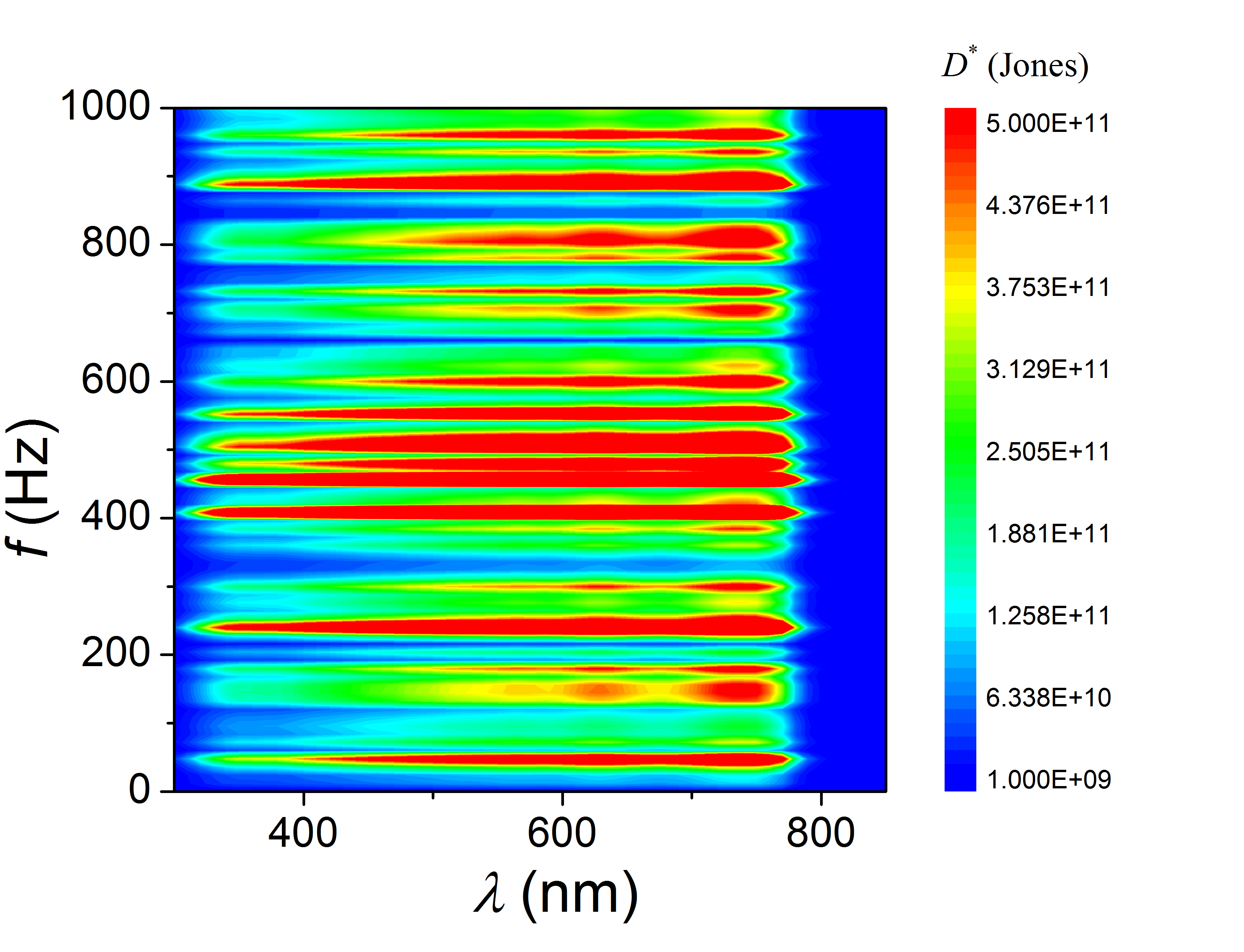

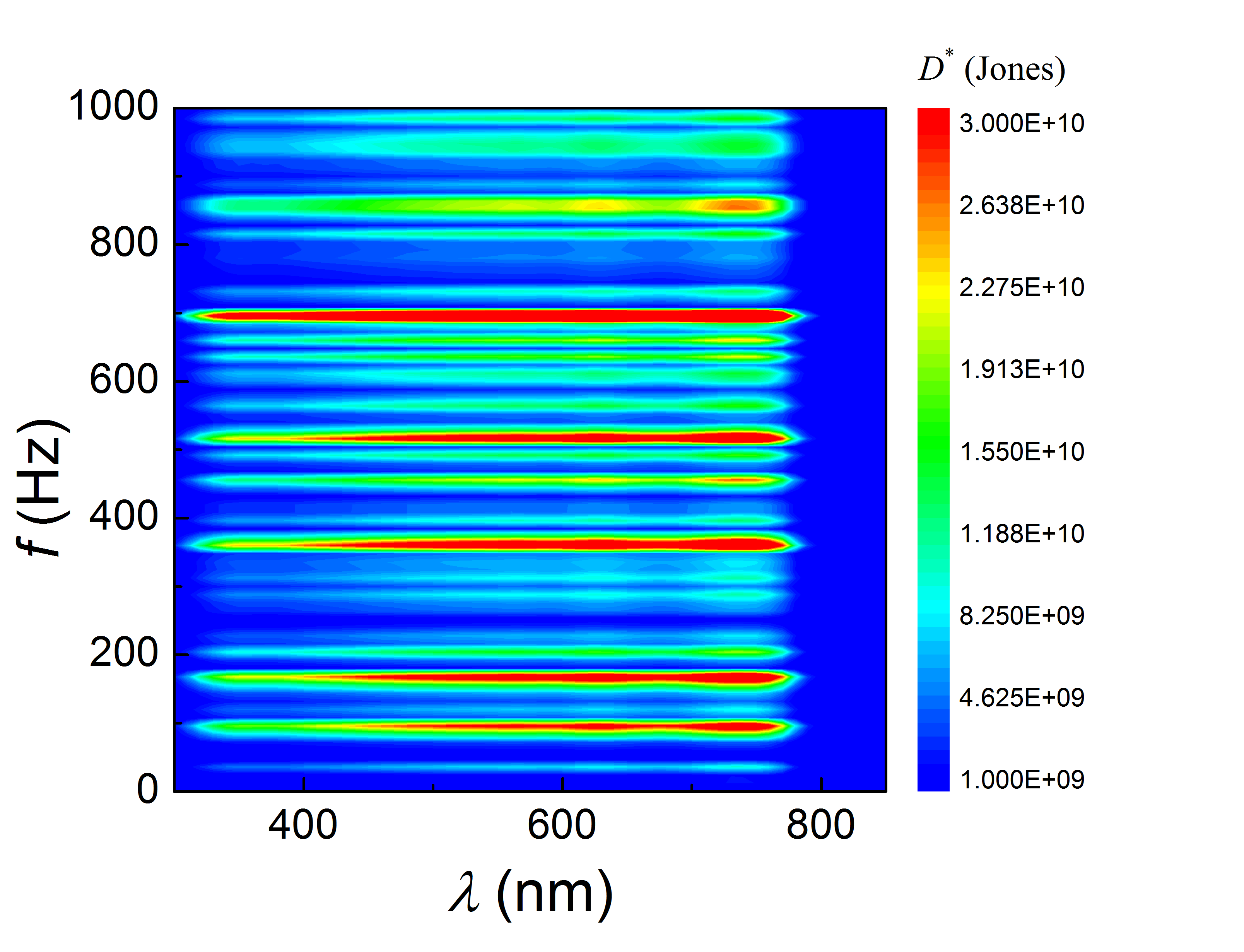
 **Figure S6.** Noise equivalent power (NEP) and specific detectivity (*D*^*^) of the devices before (left) and after (right) proton irradiation.


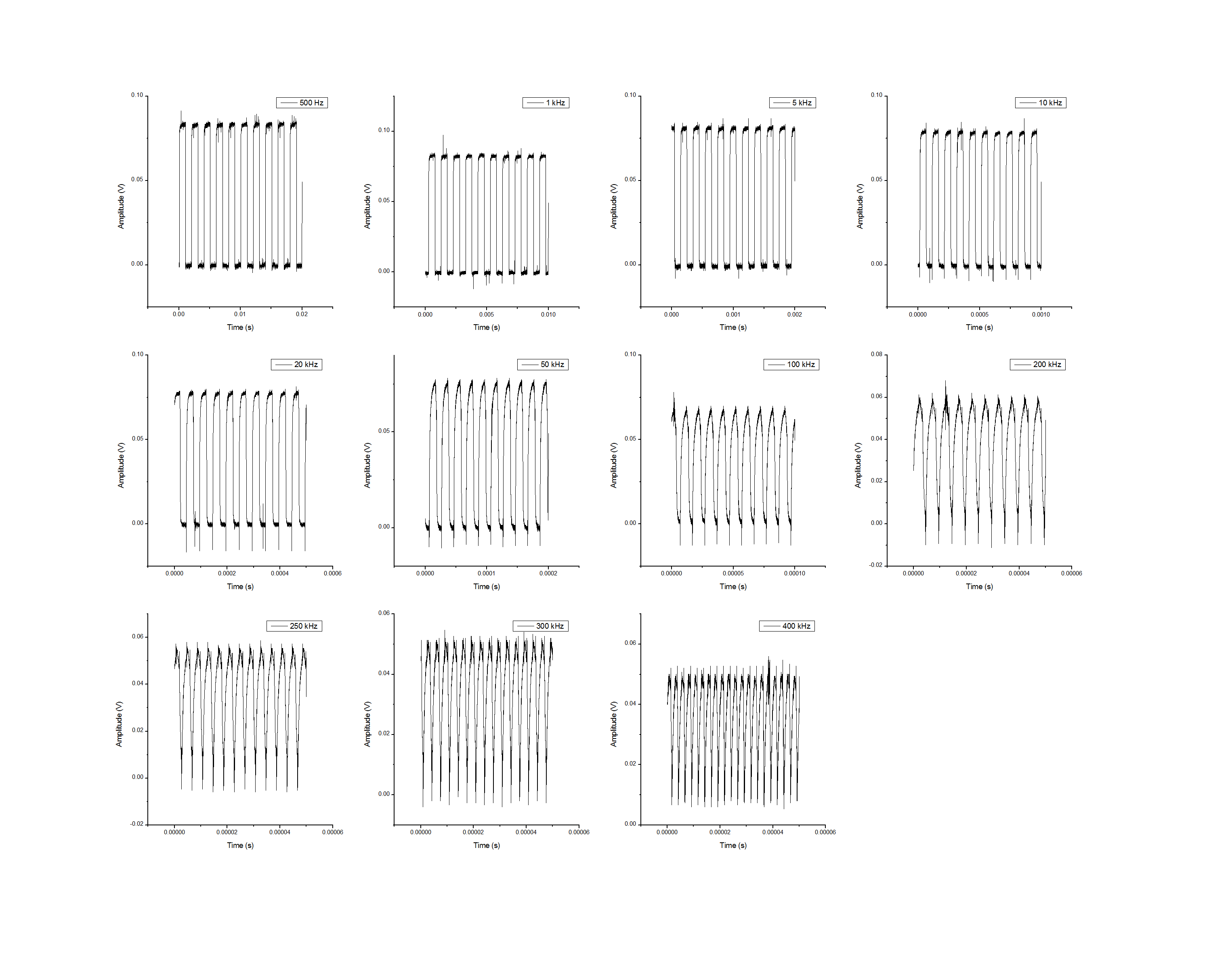


**Figure S7.** Frequency dependent responses of the tested device before proton irradiation.


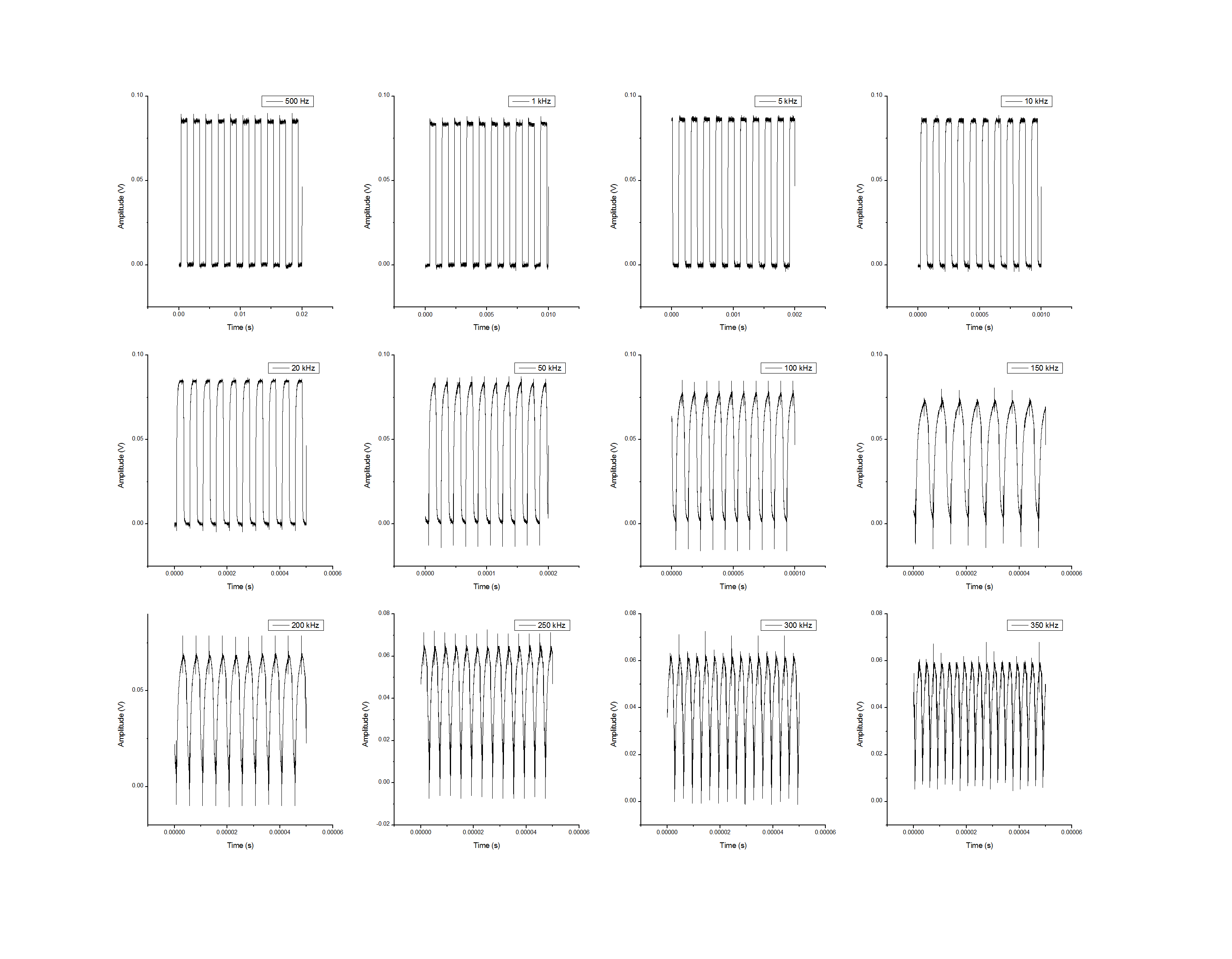


**Figure S8.** Frequency dependent responses of the tested device after proton irradiation.


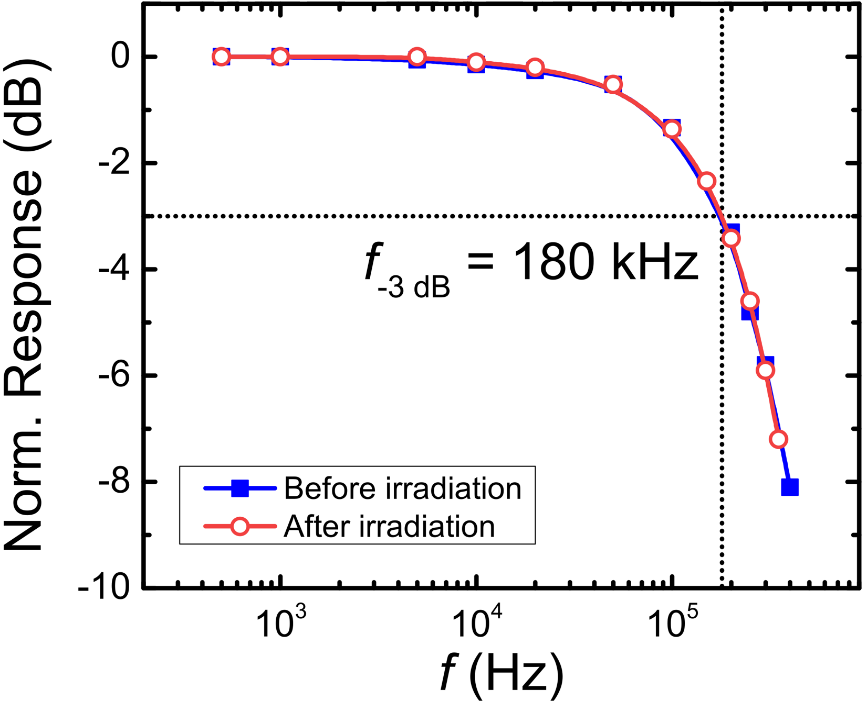


**Figure S9.** Frequency dependent normalized response of the tested devices before and after proton irradiation. The identical cutoff frequencies *f*_-3dB_ are likely caused by the limiting RC time constant.


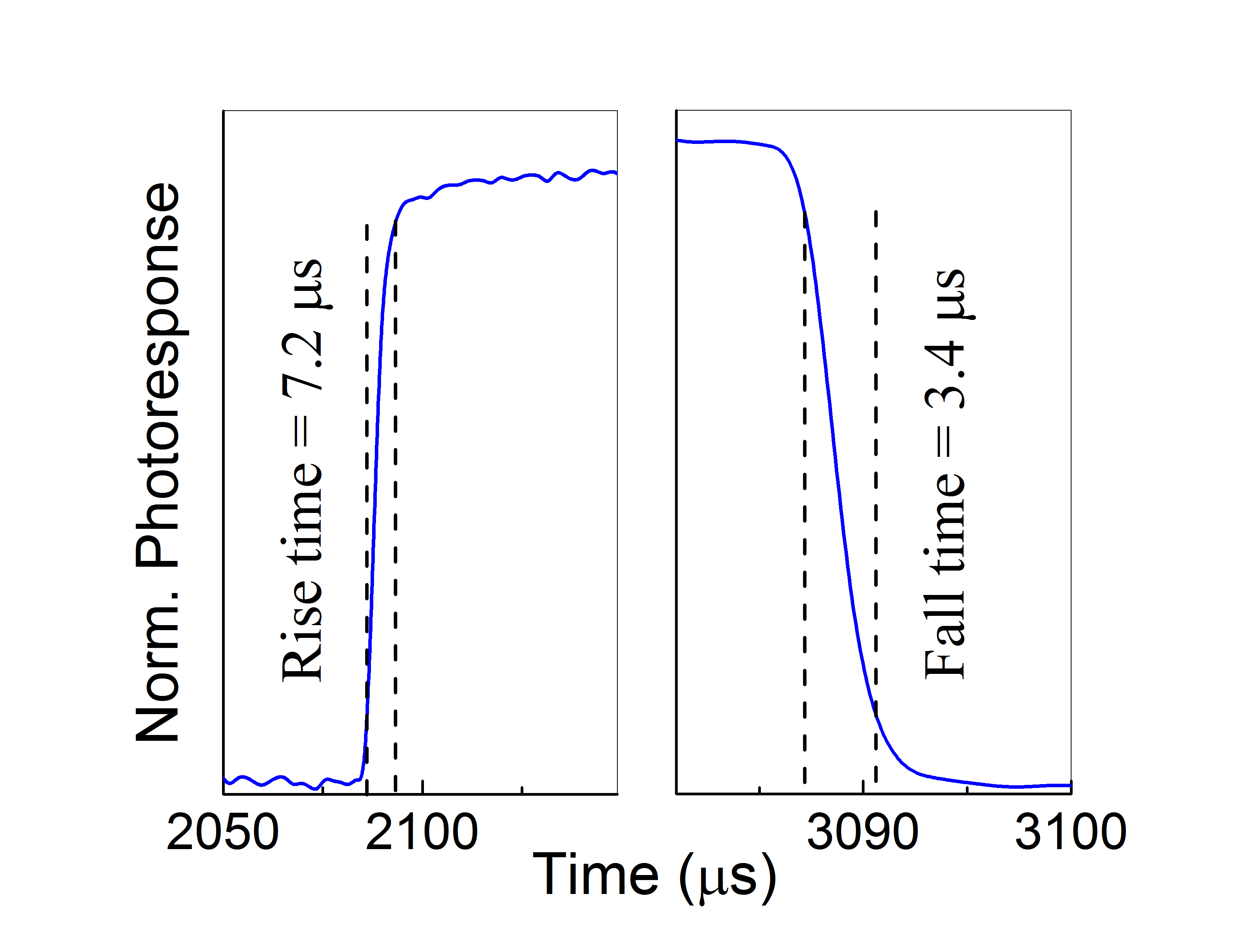

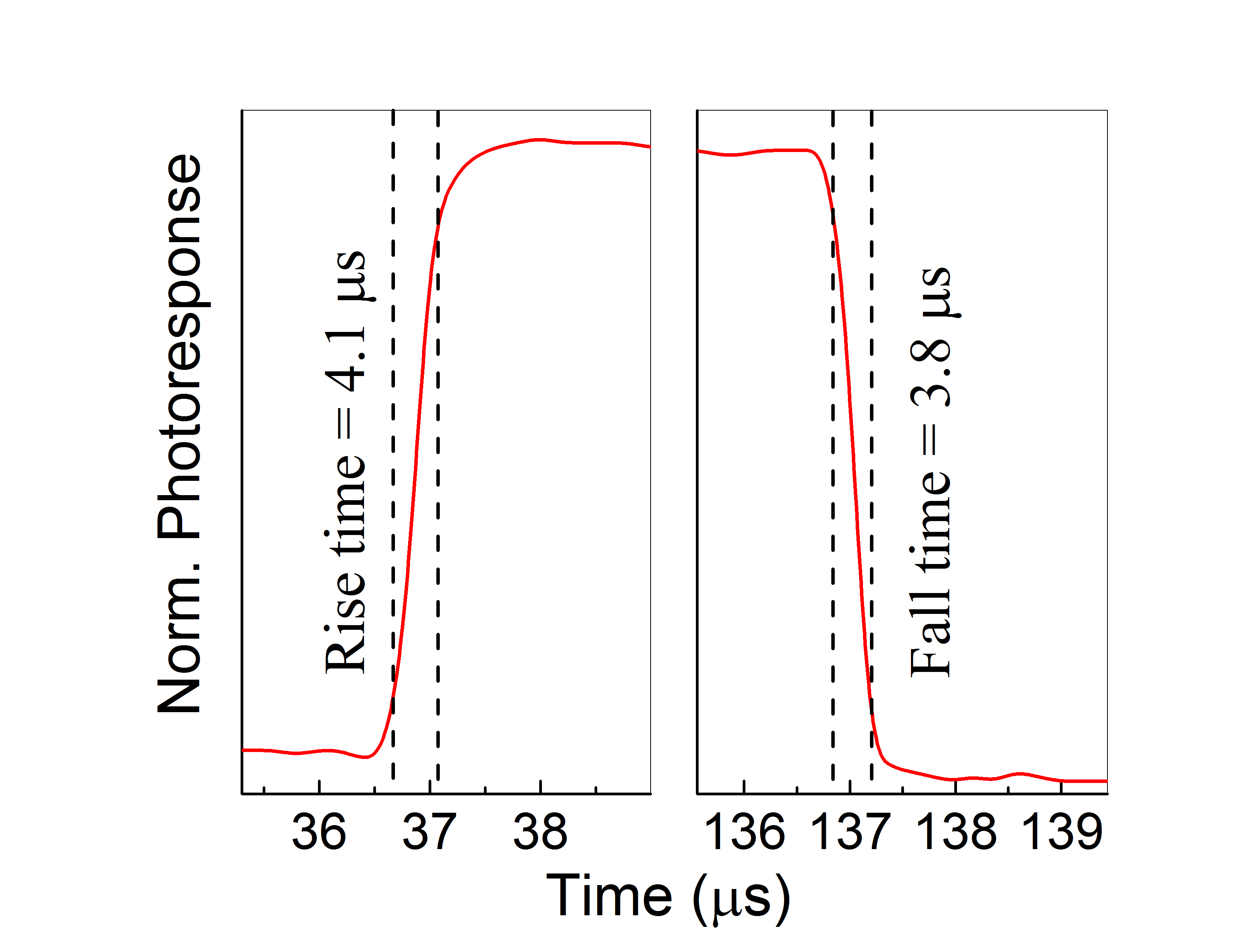


**Figure S10.** Normalized photoresponse of the tested devices before (blue) and after (red) proton irradiation. The rise and fall times are also highlighted.

**Table S1.** List of reference efficiencies and efficiencies after irradiation of perovskite solar cells studied in this work and in the literature.

| Ref. | Active Layer | *η*_ref_ [%] | *η*_irr_ [%] | *E*_p+_ [keV] | Acc. Fluence [p/cm^2^] |
| --- | --- | --- | --- | --- | --- |
| This work | CH_3_NH_3_PbI_3_ | 13.4 | 7.1 | 140 | 10^13^ |
| ^1^ | CH_3_NH_3_PbI_3_ | 12.3 | ~7.3 | 50 | 10^12^ |
| ^2^ | CH_3_NH_3_PbI_3_ | 12.1 | ~10.9 | 68000 | 10^12^ |
| ^2^ | CH_3_NH_3_PbI_3_ | 12.1 | ~7.2 | 68000 | 10^13^ |
| ^3^ | CH_3_NH_3_PbI_3_ | ~3 | ~2.8 | 150 | 10^14^ |
| ^4^ | CH_3_NH_3_PbI_3_ | 4.8 | ~5.3 | 50 | 10^14^ |
| ^5^ | CH_3_NH_3_PbI_3_ | 12.97 | ~11 | 150 | 10^15^ |
| ^4^ | FAMAPb(IBr)_3_ | 4.4 | ~3.5 | 50 | 10^14^ |
| ^6^ | FA_0.95_Cs_0.05_PbI_3_ | ~17.4 | 16.71 | 3000 | 10^13^ |
| ^7^ | Cs_0.05_(MA_0.17_FA_0.83_)_0.95_Pb(I_0.83_Br_0.17_)_3_ | 18.8 | 17.8 | 20000; 60000 | 10^12^ |
| ^8^ | Cs_0.05_(MA_0.17_FA_0.83_)_0.95_Pb(I_0.83_Br_0.17_)_3_ | 17.4 | 15.1 | 50 | 10^13^ |
| ^9^ | Cs_0.05_(MA_0.17_FA_0.83_)_0.95_Pb(I_0.83_Br_0.17_)_3_ | 15.0 | ~14.3 | 150 | 10^13^ |
| ^9^ | Cs_0.05_(MA_0.17_FA_0.83_)_0.95_Pb(I_0.83_Br_0.17_)_3_ | 15.0 | ~3.7 | 150 | 10^14^ |
| ^10^ | Cs_0.04_Rb_0.04_(FA_0.65_MA_0.35_)_0.92_Pb(I_0.85_Br_0.14_Cl_0.01_)_3_ | 18.0 | 14.8 | 170 | 2⸱10^12^ |
| ^10^ | Cs_0.04_Rb_0.04_(FA_0.65_MA_0.35_)_0.92_Pb(I_0.85_Br_0.14_Cl_0.01_)_3_ | 18.0 | 7.8 | 170 | 10^13^ |
| ^11^ | Cs_0.05_MA_0.95_PbI_3_ | 17.0 | 15.7 | 50 | 10^14^ |
| ^12^ | Cs_0.01_MA_0.01_FA_0.98_PbI_3_ | 15.5 | ~14.2 | 100 | 10^12^ |
| ^12^ | Cs_0.01_MA_0.01_FA_0.98_PbI_3_ | 15.5 | ~14.0 | 100 | 10^13^ |
| ^12^ | Cs_0.01_MA_0.01_FA_0.98_PbI_3_ | 15.5 | ~3.4 | 100 | 10^14^ |
| ^13^ | (FASn)_0.6_(MAPb)_0.4_I_3_ | 15.8 | 15.1 | 3700 | 10^11^ |
| ^14^ | Cs_0.3_FA_0.7_Pb(I_0.9_Br_0.1_)_3_ | 15.2 | 14.4 | 68000 | 2⸱10^12^ |
| ^14^ | Cs_0.3_FA_0.7_Pb(I_0.9_Br_0.1_)_3_ | 15.2 | 11.6 | 68000 | 10^13^ |
| ^15^ | InP | ~14.8 | ~11.1 | 3000 | 5⸱10^12^ |
| ^15^ | InGaP | 16.6 | ~9.4 | 3000 | 10^13^ |
| ^15^ | InGaAsP | 16.5 | ~7.7 | 3000 | 10^13^ |
| ^15^ | InGaAs | 12.5 | ~3.7 | 3000 | 10^13^ |
| ^15^ | GaAs | ~16.5 | ~6.4 | 3000 | 10^13^ |
| ^16^ | In_0.53_Ga_0.47_As | 10.71 | 4.48 | 3000 | 1.58⸱10^12^ |
| ^17^ | In_0.78_Ga_0.22_As_0.48_P_0.52_ | 14.34 | 7.52 | 3000 | 1.58⸱10^12^ |
| ^18^ | Si | 15 | 10.5 | 1000 | 10^15^ |
| ^18^ | Si | 14.1 | 11 | 1000 | 10^15^ |

**References**

1. Huang, J.-S. *et al.* Effects of Electron and Proton Radiation on Perovskite Solar Cells for Space Solar Power Application. in *2017 IEEE 44th Photovoltaic Specialist Conference (PVSC)* 1248–1252 (2017). doi:10.1109/PVSC.2017.8366410.

2. Lang, F. *et al.* Radiation Hardness and Self-Healing of Perovskite Solar Cells. *Advanced Materials* **28**, 8726–8731 (2016).

3. Miyazawa, Y. *et al.* Evaluation of radiation tolerance of perovskite solar cell for use in space. in *2015 IEEE 42nd Photovoltaic Specialist Conference (PVSC)* 1–4 (2015). doi:10.1109/PVSC.2015.7355859.

4. Miyazawa, Y. *et al.* Tolerance of Perovskite Solar Cell to High-Energy Particle Irradiations in Space Environment. *iScience* **2**, 148–155 (2018).

5. Hughes, D. *et al.* Proton Radiation Hardness of Perovskite Solar Cells Utilizing a Mesoporous Carbon Electrode. *Energy Technology* **9**, 2100928 (2021).

6. Li, P. *et al.* Tolerance of Perovskite Solar Cells under Proton and Electron Irradiation. *Materials* **15**, 1393 (2022).

7. Lang, F. *et al.* Efficient minority carrier detrapping mediating the radiation hardness of triple-cation perovskite solar cells under proton irradiation. *Energy Environ. Sci.* **12**, 1634–1647 (2019).

8. Kirmani, A. R. *et al.* Metal oxide barrier layers for terrestrial and space perovskite photovoltaics. *Nat Energy* **8**, 191–202 (2023).

9. Barbé, J. *et al.* Radiation Hardness of Perovskite Solar Cells Based on Aluminum-Doped Zinc Oxide Electrode Under Proton Irradiation. *Solar RRL* **3**, 1900219 (2019).

10. Parkhomenko, H. P. *et al.* Impact of a Short-Pulse High-Intense Proton Irradiation on High-Performance Perovskite Solar Cells. *Advanced Functional Materials* 2310404 doi:10.1002/adfm.202310404.

11. Luo, P. *et al.* Enhanced proton irradiation resistance in Cs-doped CH3NH3PbI3 films and solar cells. *Journal of Energy Chemistry* **69**, 261–269 (2022).

12. Xue, B. *et al.* Property degradation of mixed-cation perovskite films and solar cells irradiated with protons. *Nuclear Instruments and Methods in Physics Research Section B: Beam Interactions with Materials and Atoms* **526**, 29–35 (2022).

13. Durant, B. K. *et al.* Radiation stability of mixed tin–lead halide perovskites: Implications for space applications. *Solar Energy Materials and Solar Cells* **230**, 111232 (2021).

14. Lang, F. *et al.* Methylammonium-free co-evaporated perovskite absorbers with high radiation and UV tolerance: an option for in-space manufacturing of space-PV? *RSC Advances* **13**, 21138–21145 (2023).

15. Dharmarasu, N. *et al.* High-radiation-resistant InGaP, InGaAsP, and InGaAs solar cells for multijuction solar cells. *Applied Physics Letters* **79**, 2399–2401 (2001).

16. Shen, X. B. *et al.* Degradation analysis of 1 MeV electron and 3 MeV proton irradiated InGaAs single junction solar cell. *AIP Advances* **9**, 075205 (2019).

17. Xu, Y. *et al.* Spectral and electrical properties of 3 MeV and 10 MeV proton irradiated InGaAsP single junction solar cell. *Jpn. J. Appl. Phys.* **58**, 032008 (2019).

18. Ohshima, T. *et al.* Mechanism of anomalous degradation of silicon solar cells subjected to high-fluence irradiation. *IEEE Transactions on Nuclear Science* **43**, 2990–2997 (1996).
